# Metabolic sexual dimorphism in hypothalamic Fezf1 neuron-specific BDNF knockout

**DOI:** 10.1186/s13293-025-00770-z

**Published:** 2025-11-11

**Authors:** Dayana Cabral-da-Silva, Ariane M. Zanesco, Fernando Valdivieso-Rivera, Ana L. Gallo-Ferraz, Marcela R. Simões, Bruna Bombassaro, Carlos H. Sponton, Licio A. Velloso

**Affiliations:** 1 Laboratory of Cell Signaling-Obesity and Comorbidities Research Center, University of Campinas, Campinas, 13083-864 Brazil; 2 Department of Physiology, Ribeirão Preto Medical School, University of São Paulo, Ribeirão Preto, Brazil; 3 National Institute of Science and Technology on Neuroimmunomodulation, Campinas, Brazil

**Keywords:** Obesity, Diabetes, Hypothalamus, Adipose tissue, Energy expenditure

## Abstract

**Background:**

Brain-derived neurotrophic factor (BDNF) is highly expressed in the hypothalamus where it exerts regulatory functions over neurogenesis, reproduction, energy balance, and metabolism. Analyzing a hypothalamic single-nucleus transcriptomic, we identified Fezf1 ventromedial hypothalamic (VMH) neurons as an important source of BDNF. During development, Fezf1 neurons are involved in the organization of the olfactory bulb, and mutations on this gene are responsible for Kallmann syndrome; however, in adult life, little is known about the functions of Fezf1 neurons.

**Methods:**

In this study, we aimed at providing advance in the characterization of Fezf1 neurons and exploring the role of Fezf1-BDNF in the regulation of the metabolic phenotype of mice. Hypothalamic immunofluorescence was employed to determine the distribution and projections of Fezf1 neurons. Mice with a Fezf1-specific knockout of BDNF were constructed and used in the determination of the metabolic phenotype.

**Results:**

Using a Cre-Lox system to express mCherry specifically in Fezf1 neurons of the VMH, we identified projections to the dorsomedial hypothalamus and the zona incerta, regions involved in metabolic control and motor activity, respectively. The Fezf1-specific knockout of BDNF resulted in increased cold tolerance in males, and protection against diet-induced obesity due to a reduction in food intake and increased spontaneous ambulatory activity in females. This was accompanied by protection against glucose intolerance, and increased insulin sensitivity, in females.

**Conclusions:**

Thus, the present work provides advance in the understanding of the biology of VMH Fezf1 neurons, revealing the details of its distribution and projections, and demonstrating that the expression of BDNF in these neurons is involved, according to a sexual dimorphic pattern, in the regulation of metabolic function. In addition, this is the first evidence that, in a specific hypothalamic cell population, BDNF may have a detrimental rather than positive role in the regulation of systemic metabolism.

**Supplementary Information:**

The online version contains supplementary material available at 10.1186/s13293-025-00770-z.

## Highlights

- Fezf1 neurons are an important source of BDNF in the hypothalamus.
- Hypothalamic Fezf1 neurons project to dorsomedial hypothalamus and the zona incerta.
- The knockout of BDNF from Fezf1 neurons results in increased cold tolerance in males.
- The knockout of BDNF from Fezf1 neurons results in protection against diet-induced obesity due to a reduction in food intake and increased spontaneous ambulatory activity in females.

**Supplementary Information:**

The online version contains supplementary material available at 10.1186/s13293-025-00770-z.

## Abstract (plain language summary)

The hypothalamus is a brain region involved in the control of functions such as food intake, body temperature, energy expenditure, and reproduction. In this study we show that an important brain substance, namely brain derived neurotrophic factor (BDNF), is expressed in a hypothalamic subpopulation of neurons, FezF1, where it regulates body metabolism according to a sexual dimorphic pattern. In female mice, the inhibition of BDNF in hypothalamic FezF1 neurons result in protection against diet-induced obesity due to a reduction in food intake and increased spontaneous ambulatory activity, whereas in males it leads to increased cold tolerance. The study provides advance in the understanding of how the brain regulates the sexual differences in body metabolism.

**Supplementary Information:**

The online version contains supplementary material available at 10.1186/s13293-025-00770-z.

## Introduction

Brain-derived neurotrophic factor (BDNF) was first described in the hypothalamus as a component of the stress-response machinery that leads to the activation of the hypothalamic-pituitary-adrenal axis [1, 2]. Further studies demonstrated that both BDNF and its receptors are widely expressed in distinct areas of the hypothalamus suggesting they could be involved in many other functions [3–5]. This has been confirmed, as studies performed over the last 25 years have shown that BDNF is involved in the regulation of hypothalamic neurogenesis [6–8], reproduction [9], energy balance [10], and systemic metabolism [11, 12]. However, in most cases, the studies performing interventions to either increase or decrease the actions of BDNF in the hypothalamus were not targeted to specific subcellular populations [13–15]. Nevertheless, even using non-cell specific interventions, the results of those studies have placed BDNF as an important player in the hypothalamic regulation of systemic metabolism particularly by its actions in the ventromedial area (VMH).

In a preliminary phase of this study, we re-analyzed data from a mouse hypothalamus single-nucleus transcriptomics [16] as an approach to identify specific subpopulations of neurons expressing BDNF. The result showed that VMH neurons expressing Fezf1 are an important source of BDNF. The Fezf1 gene codes for a member of the zinc finger family of transcription repressors [17]. During development, Fezf1 is important for the correct penetration of axons through the olfactory plate [18], and mutations of this gene result in Kallmann syndrome, which is characterized by anosmia and hypogonadotropic hypogonadism [19]. However, little is known about the functions of Fezf1 during adult life and particularly how BDNF could be involved in these functions.

In the present study, we aimed at advancing the structural and functional characterization of the VMH Fezf1 neurons. For that, we first provided a detailed transcriptional characterization of Fezf1 neurons and an elucidation of the brain regions receiving projections from these neurons. Next, we constructed cell-specific knockout mice to explore the metabolic phenotype resulting from the ablation of Bdnf in Fezf1 neurons.

## Methods

*In silico analyses.* We reanalyzed the single-nucleus RNA-seq data from Affinati et al. [16], separating each cluster with the authors’ metadata standards in Seurat [20]. We then evaluated the expression of Fezf1 and Nr5a1 using the Nebulosa tool [21] and then all clusters with the same name were combined using Seurat’s merge tool and renamed “ident” in preparation for analysis with Seurat’s FindAllMarkers tool. After that, we performed a differential gene expression analysis between the Fezf1 cluster and the other clusters. As a result, we obtained a list of differentially expressed genes for subsequent analysis. Next, genes with significant differential expression were used as input for pathway enrichment analysis using pathFindR (version 2.3.0) [22] following the active subnetwork-driven enrichment workflow. KEGG pathways were filtered by a fold enrichment greater than two. Measurements of gene co-expression in the hypothalamus were performed using the public hypothalamic single-cell transcriptomic dataset from the Chan Zuckenberg Initiative, CellxGene plataform (https://cellxgene.cziscience.com).

*Experimental models.* Mice of the following strains were used: Fezf1-GFP (Fezf1tm1.1Bche/JrsJ #034493 Jackson Laboratory), Fezf1-cre (B6.Cg-Fezf1tm1.1(cre/folA)Hze/J - #025110 Jackson Laboratory), Bdnf-lox (B6J.129S4-Bdnftm3Jae/RujfJ #033689 Jackson Laboratory), EGFP-L10a lox (B6;129S4-Gt(ROSA)26Sortm9(EGFP/Rpl10a)Amc/J #024750 Jackson Laboratory). To obtain mice with knockout of Bdnf expression specifically in Fezf1 neurons, Fezf1-cre were crossed with Bdnf-lox mice. For immunofluorescence tests, the mice resulting from the previous cross were crossed with EGFP-L10a lox mice. All mice were kept in microisolators, in a climate-controlled room at 22 °C ± 2 with diet and water ad libitum, with 12 h/12 h light/dark cycles. Standard diets or high-fat diets (HFD) with 45% of total calories from fat were used (the compositions of diets are shown in Table 1). In the experiments aimed at obtaining tissues, mice received a lethal dose of the following mixture: ketamine hydrochloride 300 mg/kg plus xylazine hydrochloride 30 mg/kg. The experiments followed all the rules in accordance with the Ethics Committee for the Use of Animals of University of Campinas (#5591-1/2020), and by the Internal Biosafety Committee of the School of Medical Sciences (# 04/2020).

**Table 1:** Nutrient composition of diets

	HFD	Chow
	**w/w (%)**	**w/w (%)**
Starch	33.15	55
Caseine	20	22.5
Sucrose	10	0
Soy oil	4	4.5
Fibers	5	8
Mineral Mix	3.5	5
Vitamins Mix	1	5
L-Cysteine	0.3	0
Choline	0.25	0
Lard	22.8	0
Total	100	100
Kcal/g	4.99	3.57
Proteins	16.27%	21.43%
Lipids	48.34%	11.34
Carbohydrates	35.39%	67.23%

*Genotyping.* After weaning at 21 days, small fragments of the mice’s ears were collected and the DNA extracted with 100uL of 50mM NaOH at 100 °C in a dry bath for 20 min. Subsequently, it was neutralized with 10uL of 1mM Tris-HCl and centrifuged at 13,000RPM for 7 min; 2.5uL of the sample was subjected to amplification with GoTaq G2 Green Master Mix (Promega Corporation) according to the protocol indicated by Jackson Laboratory for each gene evaluated. Subsequently, the DNA bands were separated by electrophoresis in a 2% agarose gel with Sybr Safe DNA Gel Stain 10,000x (Thermo Fisher Scientific Inc).

*Cre-lox intervention.* Fezf1-cre (Fezf1-2 A-dcre-D) mice possess a destabilized Cre recombinase fusion gene adjacent to the coding region of the Fezf1 gene. This causes the dCre gene to be expressed specifically by the endogenous Fezf1 expression promoters. The ecDHFRR12Y/Y100I domain of dCre leads to proteasomal degradation of the entire fusion protein, resulting in reduced Cre recombinase activity. Administration of the DHFR inhibitor trimethoprim (TMP) prevents degradation of the dCre fusion gene and results in increases in Cre recombinase activity. When Fezf1-cre mice are crossed with mice containing loxP-flanked sequences, TMP-enabled Cre recombination results in deletion of the floxed sequences only in the Fezf1-expressing cells of the offspring. The dose of TMP (Trimethoprim T7883 Sigma Aldrich) used was 0.30 mg/g of animal, solubilized in saline immediately before administration by gavage for three consecutive days, always in the sixth week of life. The animals were only used for experiments after the sixth week of life, having a minimum of two weeks of Cre-recombinase expression.

*Stereotaxic intervention.* All mice used were at least 8 weeks old and were anesthetized with a mixture of ketamine (100 mg/kg of animal) and xylazine (10 mg/kg of animal) diluted in saline. Surgery was performed with a digital stereotaxic device (Bonther) and a 1 uL borosilicate pipette (1 µL, Model 7001 Knurled Hub Hamilton) positioned for injection of viral vectors. Injections were performed in the VMH region (coordinates AP −1.34, L ± 0.3, and − 5.6 mm from bregma)88 bilaterally and the injected volume was 100 nL. After injection, the animals remained for 5 min with the needle in position to avoid extravasation of the liquid. After surgery, the animals were analgesic with tramadol (25 mg/kg of animal) and remained under observation for at least 2 h before returning to the microisolator racks. A minimum of two weeks elapsed between surgery and the experiments to ensure sufficient expression of the proteins of interest.

*Fezf1 neuron projections.* Neuronal projections were evaluated with particles of the pAAV-hSyn-DIO-mCherry-AAV1 50,459 vector (titer 1.7 × 1013) obtained from Addgene; 100nL of viral particles were administered through stereotaxic surgery bilaterally in the region of the ventromedial nucleus of the hypothalamus. Cre recombinase expression was induced by TMP in the sixth week of life and stereotaxic surgery was performed in the eighth week; brain extraction was performed two weeks after surgery.

*Determination of interscapular temperature and brown adipose tissue morphology.* An infrared camera (IR) (FLIR T450sc, FLIR Systems, Inc. Wilsonville, USA) with an IR resolution of 320 × 240 pixels was used. The images obtained were presented in high-contrast rainbow mode, which is available in the color palette of the free FLIR Tools IR software. Temperature measurements were performed with the FLIR Tools software using the circle tool with an ROI of 20. The maximum temperatures of each measurement were recorded, and normalization was performed with the temperature obtained using the eye as a region of interest. At the end of experiments, the brown adipose tissue (BAT) was removed and submitted to macroscopic and microscopic evaluation.

*Respirometry measurements.* O2 consumption, CO2 production and Respiratory Quotient (RQ) were determined for 24 h using a LE405 Gas Analyzer (Panlab – Harvard Apparatus, Holliston, MA, USA). The mice were previously acclimated in the respirometric chambers 24 h before the beginning of the measurements. The air flow was maintained by an Air Supply & Switching flowmeter (Panlab – Harvard Apparatus, Holliston, MA, USA) and the gas analyzer previously calibrated with known concentrations of O2 and CO2 (Air Liquid, São Paulo, Brazil). The 3-minute recordings for each animal were made every 30 min, and the room air was used as a reference. Air samples from the chambers and from the room passed sequentially through the O2 and CO2 sensors to determine the concentration of the gases and measure oxygen consumption and carbon dioxide production. The O2 and CO2 flux (VO2 and VCO2) were calculated by the Metabolism 2.2v program and expressed in mL.h-1, based on the Withers equation; and the RQ was calculated using VCO2/VO2.

*Locomotor activity.* Locomotor activity was determined for 24 h using the Le001 PH Multitake Cage (Panlab Harvard Apparatus, Holliston, MA, USA) and was recorded and computed using Compulse v1.0 software (Panlab Harvard Apparatus, Holliston, MA, USA). Mice were acclimated in the chambers for 24 h before the start of the measurements. The recordings were performed every second and the sums of the movements in the x plane were counted for a total of 24 h, as well as for the light (6 am to 6 pm) and dark (6 pm to 6 am) periods.

*Glucose tolerance test.* Mice fasted for 12 h during the dark cycle, after which they were re-fed for 30 min and fasted again for 4 h. Fasting blood glucose was measured and then the mice received a dose of 2.5 g/kg of glucose intraperitoneally. Blood glucose was measured by a drop of blood from the animal’s tail using a conventional glucometer at times 0, 15, 30, 60, 90 and 120 min.

*Insulin tolerance test.* Mice fasted for 12 h during the dark cycle, after which they were re-fed for 30 min and fasted again for 2 h. Fasting blood glucose was measured and then mice received a dose of 1.5 U/kg of animal of regular insulin diluted in saline intraperitoneally. Blood glucose was measured by a drop of blood from the animal’s tail using a conventional glucometer at times 0, 5, 10, 15 and 20 min. Glucose tolerance tests and insulin tolerance tests were always performed in the same sets of mice; glucose tolerance tests were performed first and insulin tolerance tests were performed one week later.

*Quantitative real-time PCR.* Total RNA extraction from the hypothalamus and the BAT was performed using the Trizol reagent method (Invitrogen Corporation, CA, USA). For cDNA production, the High-Capacity cDNA Reverse Transcription Kit (Applied Biosystems, Foster City, CA, USA) was used, with a final cDNA concentration of 2.0 ug. This cDNA was diluted according to the concentration required for efficient amplification of each gene. Real-time PCR reactions were performed in the QuantStudio™ 6 Flex Real-Time PCR System (Applied Biosystems) using the TaqManTM system (Applied Biosystems). Cycles were 95 °C for 2 min followed by 40–45 cycles of 95 °C for 15 s and 60 °C for 60 s. The GAPDH Mouse (Applied Biosystems), Hprt Mouse (Applied Biosystems) or 18 S Mouse (Applied Biosystems) gene were used as endogenous control of the reaction, normalizing the expression of the gene of interest in the different samples.

*Histological analysis using immunofluorescence.* Immunofluorescence was used to analyze the tissue distribution of Fezf1 neurons and to determine the co-expression of several proteins in this particular subpopulation of neurons. The mice were anesthetized and perfused with a trans-cardiac injection of 0.9% saline solution followed by 4% paraformaldehyde (PFA). The brains were removed and immersed for 24 h in PFA, and then immersed in 30% sucrose solution until the tissues sank. The brains were then sectioned in coronal or sagittal 30 μm specimens using a cryostat (LEICA Microsystems CM1860, Buffalo Grove, IL, USA). The sections were kept in antifreeze solution at −20 °C until the immunofluorescence assay was performed. The tissues were washed three times for 5 min in PBS 1X, then permeabilized with a 0.15% hydrogen peroxide solution for 10 min. After rinsing, the sections were blocked for 2 h in a PBS1x + 0.2% Triton 10% + 5% goat serum solution and then incubated with the primary antibodies overnight. The following day, the sections were washed again and incubated with secondary antibodies for 2 h. The nuclei were stained with DAPI or To Pro 3. Fluorescence images were obtained with an Upright LSM780-NLO Zeiss confocal microscope. For the projection images, a mosaic was created with the Spinning Disk confocal microscope - ANDOR Technology.

*Statistical analysis.* All results were expressed as mean ± standard error of the mean. First, the presence of outliers was verified using the ROUT test with Q of 5%; after removing possible outliers, the normality of the samples was verified using the Shapiro-Wilk normality test. Once the normal distribution was observed, the Student’s *t*-test was used; if normality was not verified, the respective nonparametric Mann-Whitney test was performed. In some experiments we used one-way ANOVA; one-way was chosen because males and females were always evaluated separately, thus, presence or absence of Bdnf in Fezf1 neurons was the only variable under evaluation. The significance level was set at *p* ≤ 0.05, and data analysis was performed using GraphPad Prism 10.3 software.

## Results

**Fig. 1:**
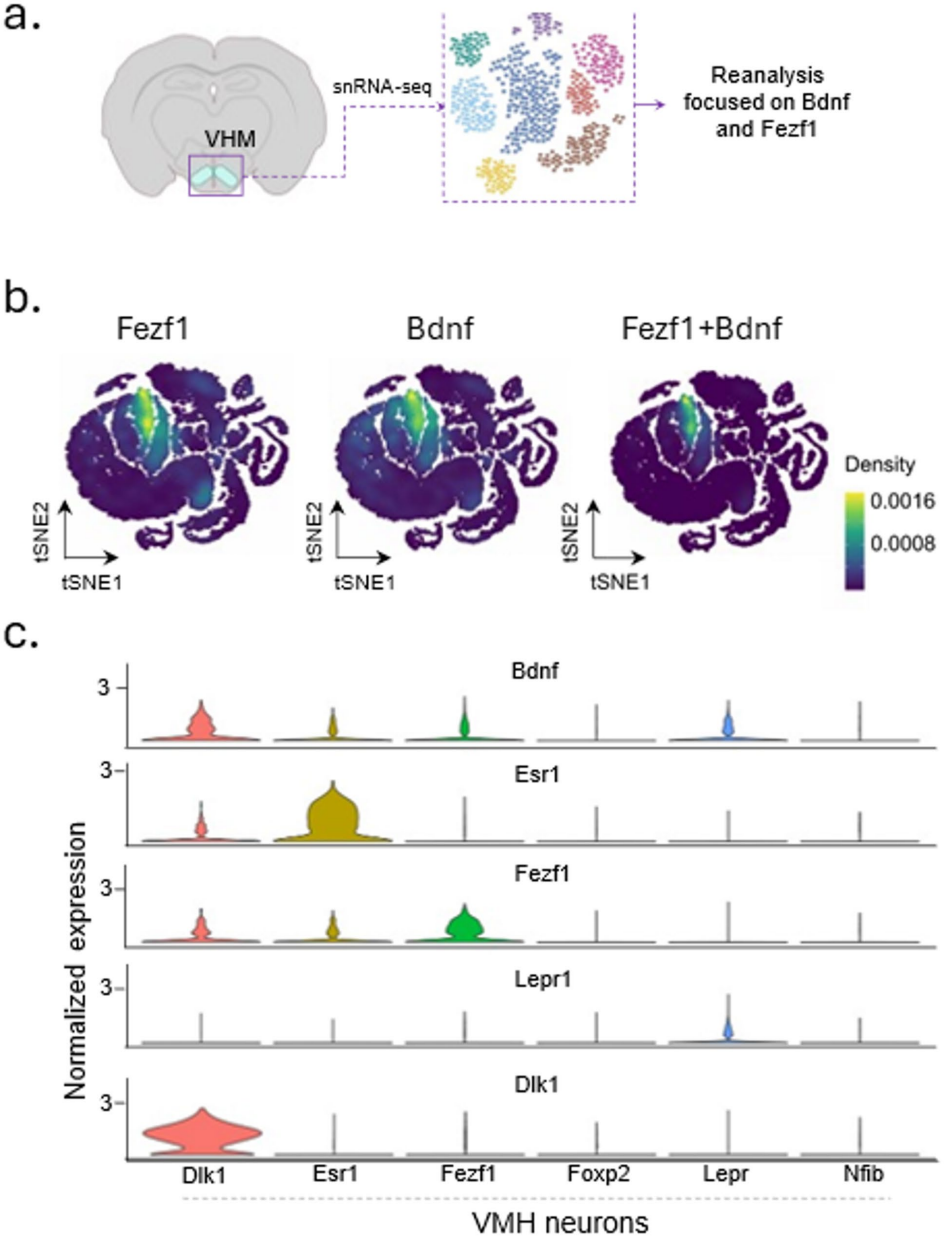
Single-nucleus RNA sequencing of cells from the ventromedial hypothalamus identifies subpopulations expressing Bdnf in the mouse brain. Data from Affinati and coworkers (DOI: 10.7554/eLife.69065) were re-analyzed with focus on the expression of BDNF. In the original study, cells from the ventromedial hypothalamus (VMH) were harvested and submitted to single-nucleus RNA sequencing (**a**). Cell clusters expressing Fezf1, Bdnf and the co-expression Fezf1 + Bdnf are depicted in (**b**). Violin-plots depict the expression levels of Bdnf, Esr1, Fezf1, Lepr1, and Dlk1 in cell clusters according to the original notations of Affinati and coworkers (**c**)

*Fezf1 neurons are an important source of BDNF in the hypothalamus.* To determine the main subpopulations of VMH neurons expressing Bdnf, we re-analyzed data from Affinati and coworkers [16] (Fig. 1a). First, we identified a considerable overlap in the cells expressing Fezf1 and Bdnf (Fig. 1b). Next, using the same notations of subpopulations defined by the original authors, we identified Fezf1 neurons as the second major source of Bdnf transcripts in the VMH (Fig. 1c). The greatest amount of Bdnf transcripts were found in Dlk1 neurons. Esr1 and Lepr neurons expressed Bdnf in amounts slightly smaller than Fezf1, whereas Foxp2 and Nfib neurons expressed even smaller amounts of Bdnf.

**Fig. 2:**
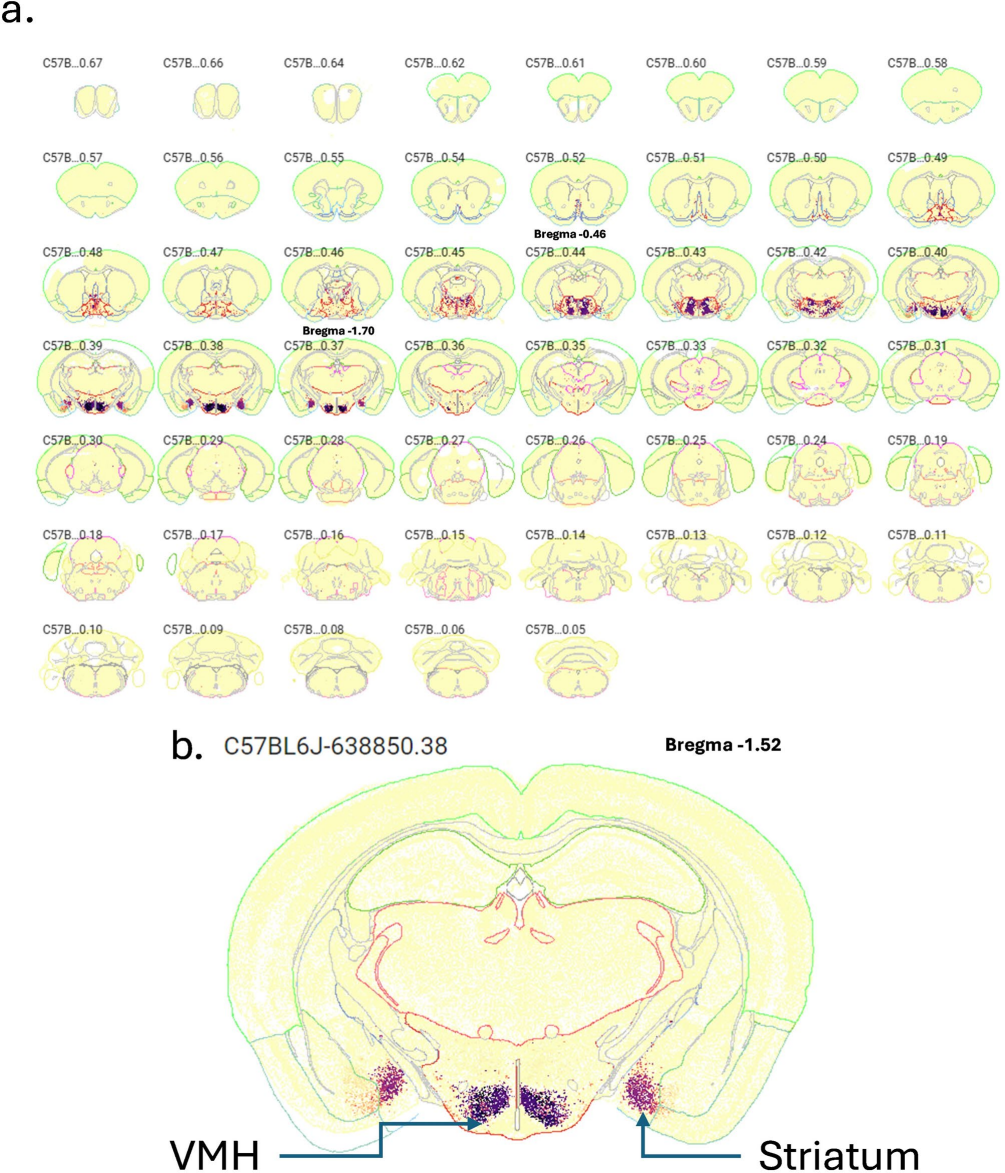
Fezf1-expressing cells in the mouse brain. The Allen Brain ABC Mouse Brain Atlas (knowledge.brain-map.org/abcatlas) was employed to determine the anatomical distribution of cells expressing Fezf1 in the whole brain. In **a** each panel corresponds to one sequencial coronal section, the greatest expression of Fezf1 occurred between Bregma − 0.46 and − 1.70, as depicted in the respective images. In **b** high-magnification image depicting the expression of Fezf1 in the ventromedial hypothalamus (VMH) and striatum

**Fig. 3:**
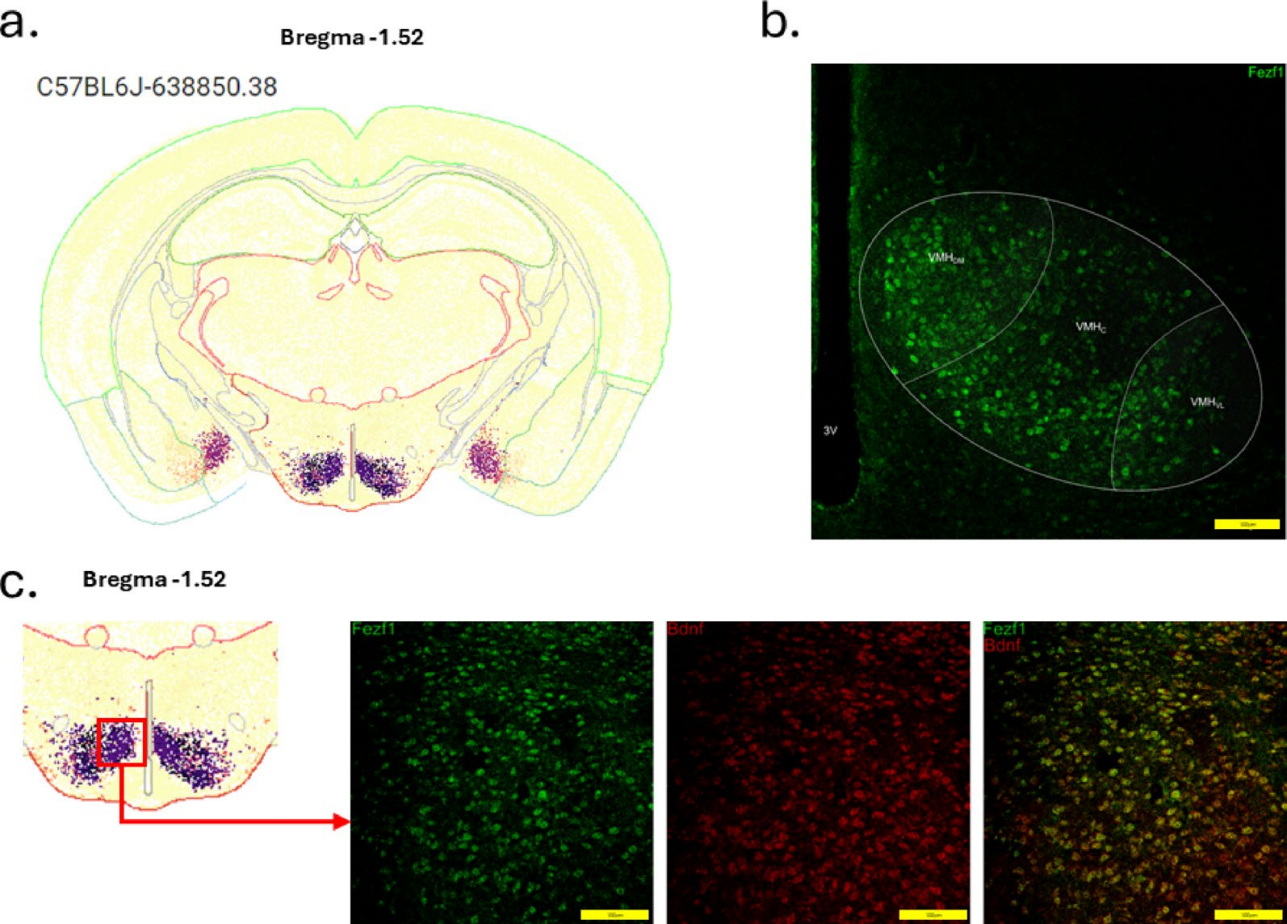
Fezf1 and BDNF expression in the ventromedial hypothalamus. In (**a**), a coronal section at Bregma − 1.52 depicts the region of the mouse brain with the greatest number of cells expressing Fezf1. In (**b**), confocal imaging of the ventromedial hypothalamus of Fezf1 reporter mice, the coronal section was obtained at Bregma − 1.52. In (**c**), sections obtained at Bregma − 1.52 from Fezf1 reporter mice were employed in immunofluorescence staining for BDNF. In (**b** and** c**), the images are representative of three independent experiments. In b and c, Fezf1, green; BDNF, red. Images in b and c are representative of three experiments, *n* = 3

**Fig. 4:**
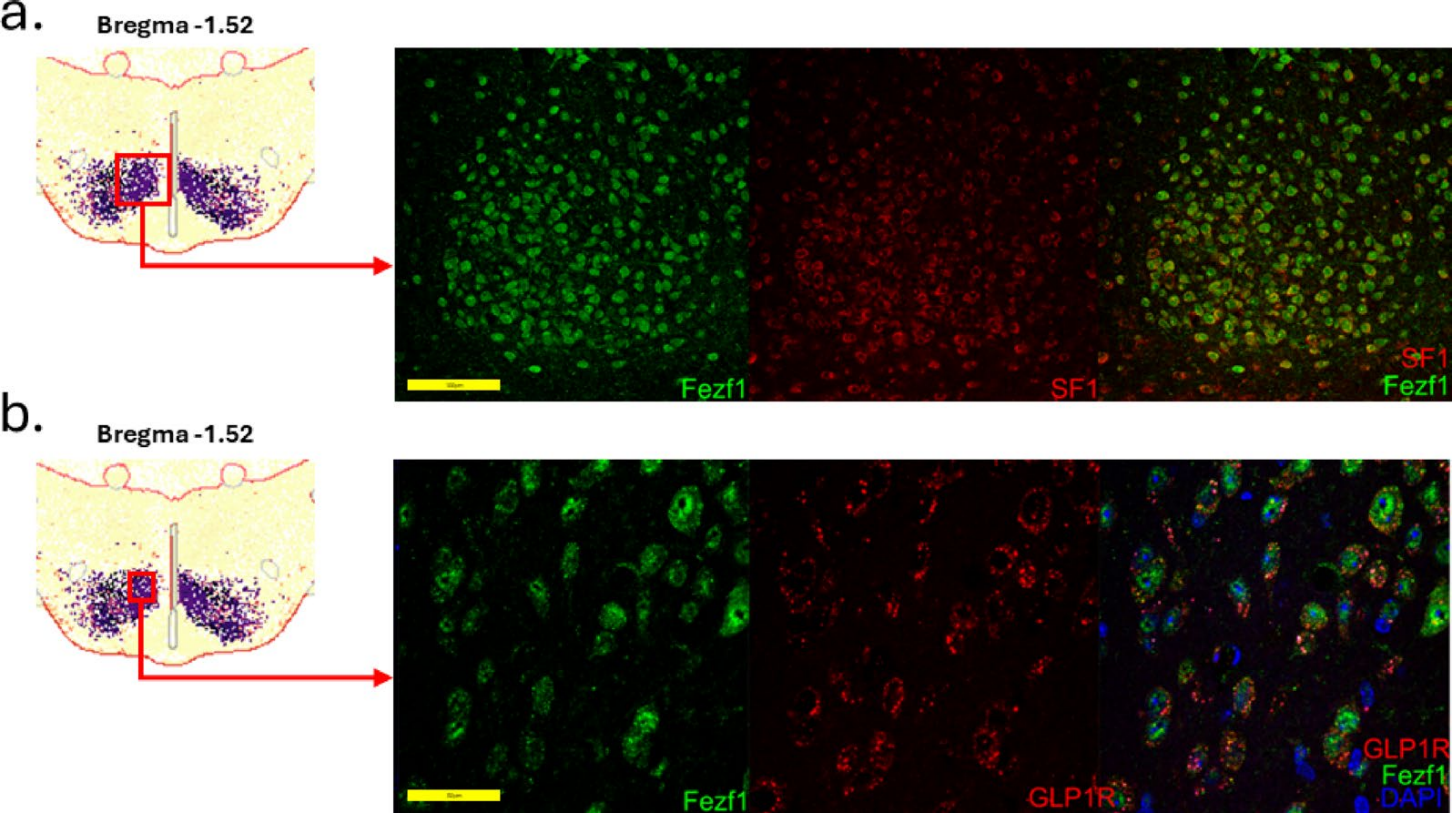
Co-expression of Fezf1 with steroidogenic factor 1 and the receptor of glucagon-like peptide 1 in the mouse brain. Sections obtained at Bregma − 1.52 from Fezf1 reporter mice were employed in immunofluorescence staining for steroidogenic factor 1 (SF1) (**a**) and the receptor of glucagon-like peptide 1 (GLP1R). In (**a** and **b**), the images are representative of three independent experiments. In a, Fezf1, green; SF1, red. In b, Fezf1, green; GLP1R, red. Images are representative of three experiments, *n* = 3

*Fezf1 neuron distribution.* Using the Allen Brain ABC Mouse Brain Atlas (knowledge.brain-map.org/abcatlas), we identified Fezf1 neurons in the hypothalamus, particularly in the VMH and in the striatum (Figs. 2a and b and 3a). In Fig. 2, the greatest expression of Fezf1 occurs in coronal sections beginning at Bregma − 0.46 up to Bregma − 1.70. In a caption of coronal section Bregma − 1.52, the expression of Fezf1 in the VMH and striatum is depicted in detail. Next, we employed Fezf1 reporter mice to determine the distribution of Fezf1 neurons in the hypothalamus. As shown in Fig. 3b, Fezf1 neurons are present in large numbers, particularly in the dorsomedial portion of the VMH. Virtually no Fezf1 neurons were found in the arcuate nucleus (ARC), the lateral hypothalamus, and the periventricular nucleus. In immunofluorescence staining, we confirmed the data obtained in single-nucleus transcriptomics showing that most VMH-Fezf1 neurons express BDNF (Fig. 3c). We also showed that some (63%), but not all Fezf1 neurons express SF1 (Fig. 4a and Suppl. Figure 1a), which is an important marker of the VMH; moreover, most (86%) Fezf1 neurons express the receptor for glucagon-like peptide-1 (GLP1) (Fig. 4b and Suppl. Figure 1b), which is an important target for first line drugs used in the treatment of diabetes and obesity.

**Fig. 5:**
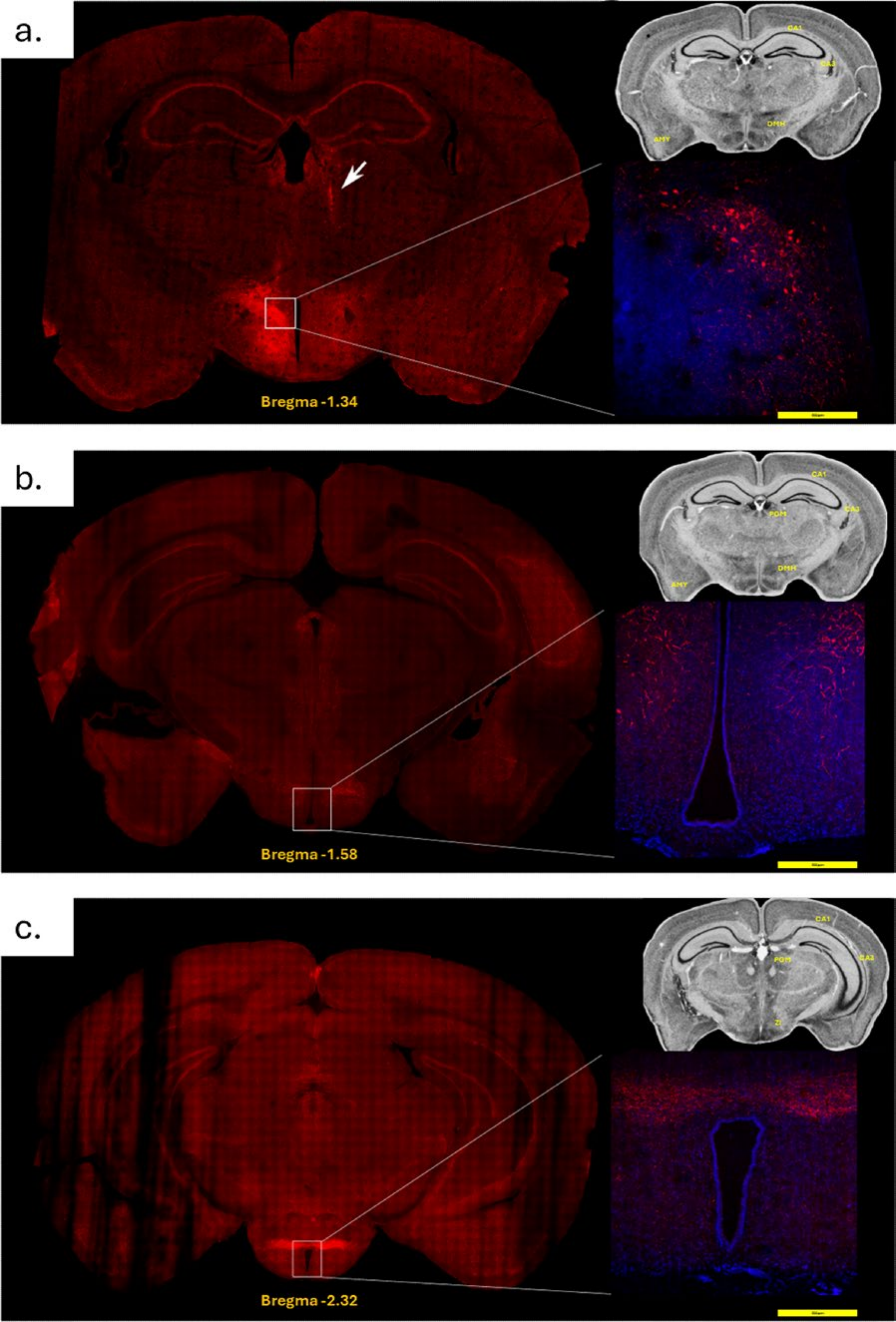
Projections from Fezf1 neurons. Fezf1 projections were determined in confocal microscopy of mouse brain sections obtained from Fezf1-cre (Fezf1-2 A-dcre-D) mice submitted to an intracerebroventricular injection of viral particles pAAV-hSyn-DIO-mCherry-AAV1 50,459. Whole brain coronal sections were obtained from Bregma − 1.34 (**a**), −1.58 (**b**), and − 2.32 (**c**). The images are representative of three independent experiments. In (**a**), the arrow indicates the path of the injecting needle. Images are representative of three experiments, *n* = 3. AMY, amygdala; CA1 and CA3, hippocampus regions; DMD, dorsomedial hypothalamus; PGM, periaqueductal gray matter; ZI, zona incerta. The anatomical reference guides depicted in the right-hand upper corner of each panel were obtained from the public dataset of The Mouse Brain Library, University of Tennessee (www.braininfo.rprc.wahington.edu)

*Fezf1 neuron projections.* Fezf1-cre (Fezf1-2 A-dcre-D) mice were submitted to an intracerebroventricular (icv) injection of viral particles pAAV-hSyn-DIO-mCherry-AAV1 and the Fezf1 projections were determined. Within the hypothalamus, projections occurred mostly to the dorsomedial hypothalamus (DMH) (Fig. 5a and b), and to the zona incerta (ZI) (Fig. 5c). Extra-hypothalamic projections occurred to the periaqueductal gray matter (Fig. 5b and c), amygdala (Fig. 5a and b), and hippocampal regions CA1 and CA3 (Fig. 5a and c).

**Fig. 6:**
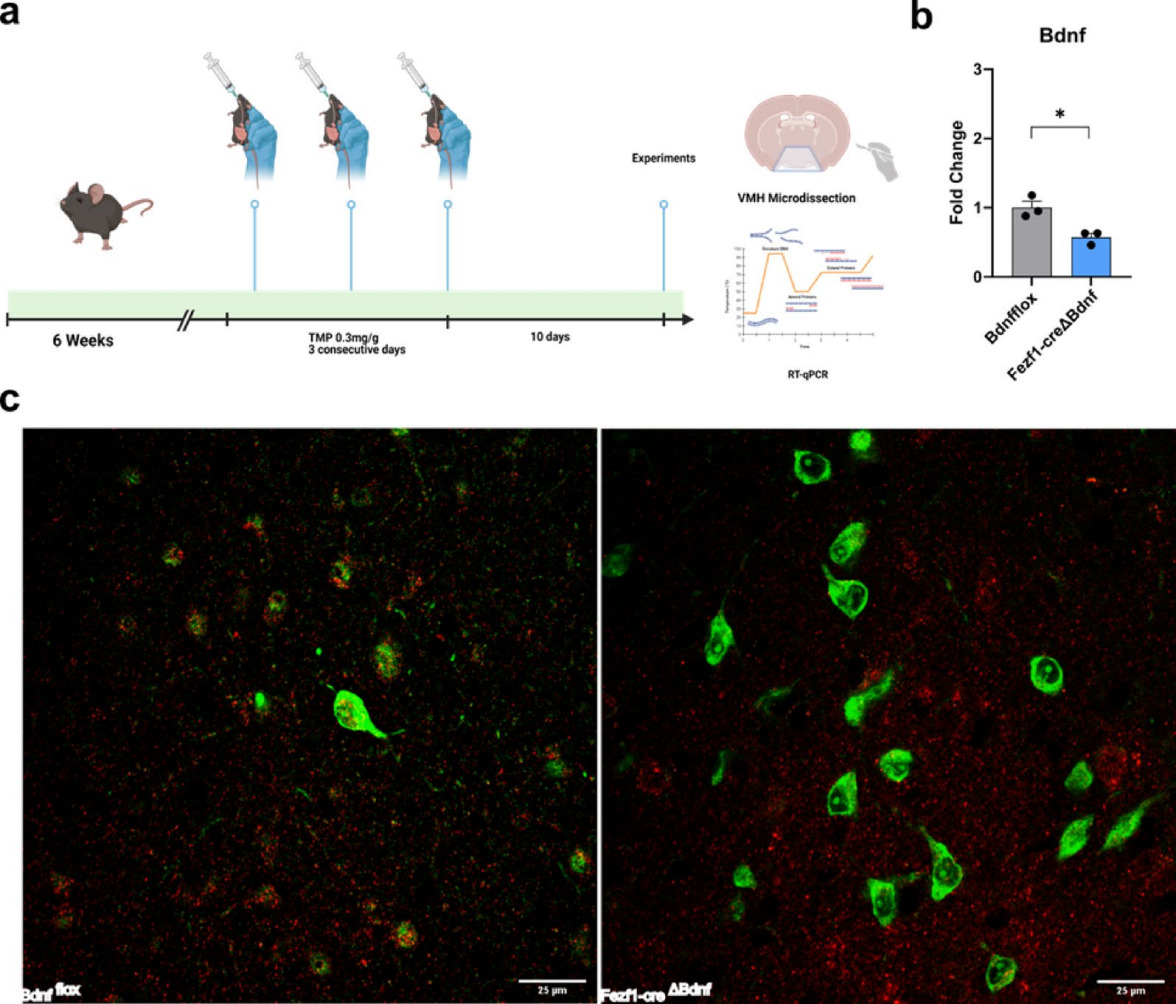
Loss-of-function of Bdnf in Fezf1 neurons. Six-week-old Fezf1-cre/EGFP-L10aΔBdnf (Fezf1-Bdnf knockout) mice were treated with three doses of trimethoprim (TMP) and after 10 days the experiments were performed (**a**). Expression of Bdnf was determined in dissected ventromedial hypothalamus by quantitative realtime PCR (**b**) and immunofluorescence of histological sections (**c**). In all experiments, n=3; *p < 0.05; in c, Fezf1 (green), BDMF (red).

**Fig. 7:**
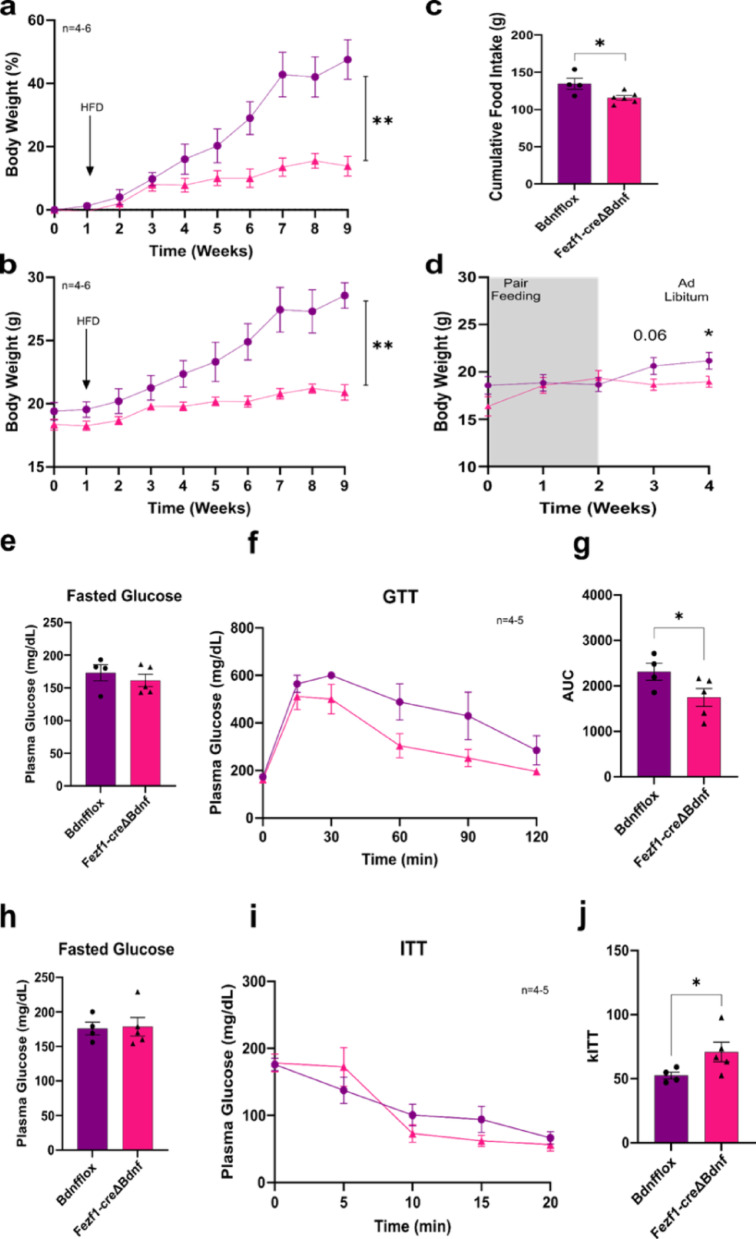
The metabolic outcomes of knocking BDNF out of Fezf1 neurons in female mice fed on a high-fat diet. All experiments were performed with Fezf1-cre/EGFP-L10a∆Bdnf (Fezf1-Bdnf knockout) female mice. Weekly determination of body weight (in % of initial mass) (**a**), and (in g) (**b**). Food intake over a period of nine weeks (**c**); body weight (in g) during a period of pair feeding (**d**). Blood levels of glucose in fasting mice (**e**); graphic representation of blood glucose levels during a glucose-tolerance test (**f**); area under the curve (AUC) obtained from the blood glucose level variations during the glucose tolerance test (**g**). Blood levels of glucose in fasting mice (**h**); graphic representation of blood glucose levels during an insulin tolerance test (**i**); graphic representation of the constant of the blood glucose disappearance rate (kITT) obtained from the blood glucose level variations during the insulin tolerance test (**j**). Bdnf-flox (purple, Bdnfflox, control) and Fezf1-cre/EGFP-L10a∆Bdnf (pink, Fezf1-creDBdnf, Fezf1-Bdnf knockout). In all experiments, *n* = 4–6; *p < 0.05

*Inhibition of Fezf1 Bdnf protects against diet-induced obesity in female mice.* Male and female Fezf1-cre/EGFP-L10a∆Bdnf (Fezf1-Bdnf knockout) mice were treated for three consecutive days with trimethoprim for inducing the conditional deletion of Bdfn specifically in Fezf1 neurons (Fig. 6a). The intervention resulted in 50% reduction of hypothalamic Bdnf expression (Fig. 6b) and virtually eliminated all BDNF expression in Fezff1 neurons of the VMH (Fig. 6c). In male mice fed either chow (Suppl. Figure 2–5) or a high-fat diet (HFD) (Suppl. Figure 6–9), the intervention resulted in no changes in body mass gain (Suppl. Figure 2a-2b and 5a-6b), food intake (Suppl. Figure 2c and 6c), glucose tolerance (Suppl. Figure 3a-3c and 7a-7c), insulin sensitivity (Suppl. Figure 4a-4c and 8a-8c), energy expenditure (Suppl. Figure 5a-5d and 9a-9d), and spontaneous locomotor activity (Suppl. Figure 5e and 9e). Likewise, in female mice fed on chow, the intervention of knocking out Bdnf from Fezf1 neurons resulted in no changes in body mass gain (Suppl. Figure 10a-10b), food intake (Suppl. Figure 10c), glucose tolerance (Suppl. Figure 11a-11c), and insulin sensitivity (Suppl. Figure 12a-12c); however, energy expenditure was significantly reduced (Suppl. Figure 13a-13d), whereas there was no change in spontaneous locomotor activity (Suppl. Figure 13e). In female mice fed in HFD, the intervention resulted in a protection against diet-induced obesity, as depicted in graphs showing relative (Fig. 7a) and actual (Fig. 7b) body mass changes, and this was accompanied by reduced food intake (Fig. 7c). Because mutant females were protected from diet-induced obesity, we reanalyzed the insulin and glucose tolerance tests, at this time correcting the results for body mass, nevertheless, the results persisted. To explore the impact of food intake reduction on the obesity protection phenotype, we performed a two-week pair-feeding experiment, and as shown in Fig. 7d, during the intervention, control mice maintained body mass similar to Fezf1-Bdnf knockout, whereas, as early as two weeks after retuning to *ad libitum* feeding, the body mass was significantly increased.

*Improved glucose tolerance and increased insulin sensitivity in female Fezf1-Bdnf knockout mice fed on a HFD.* In female mice fed on a HFD, fasting blood glucose levels were not affected by the knockout of BDNF in Fezf1 neurons (Fig. 7e); nevertheless, there was greater glucose tolerance (Fig. 7f and g) and greater insulin sensitivity (Fig. 7h and j).

**Fig. 8:**
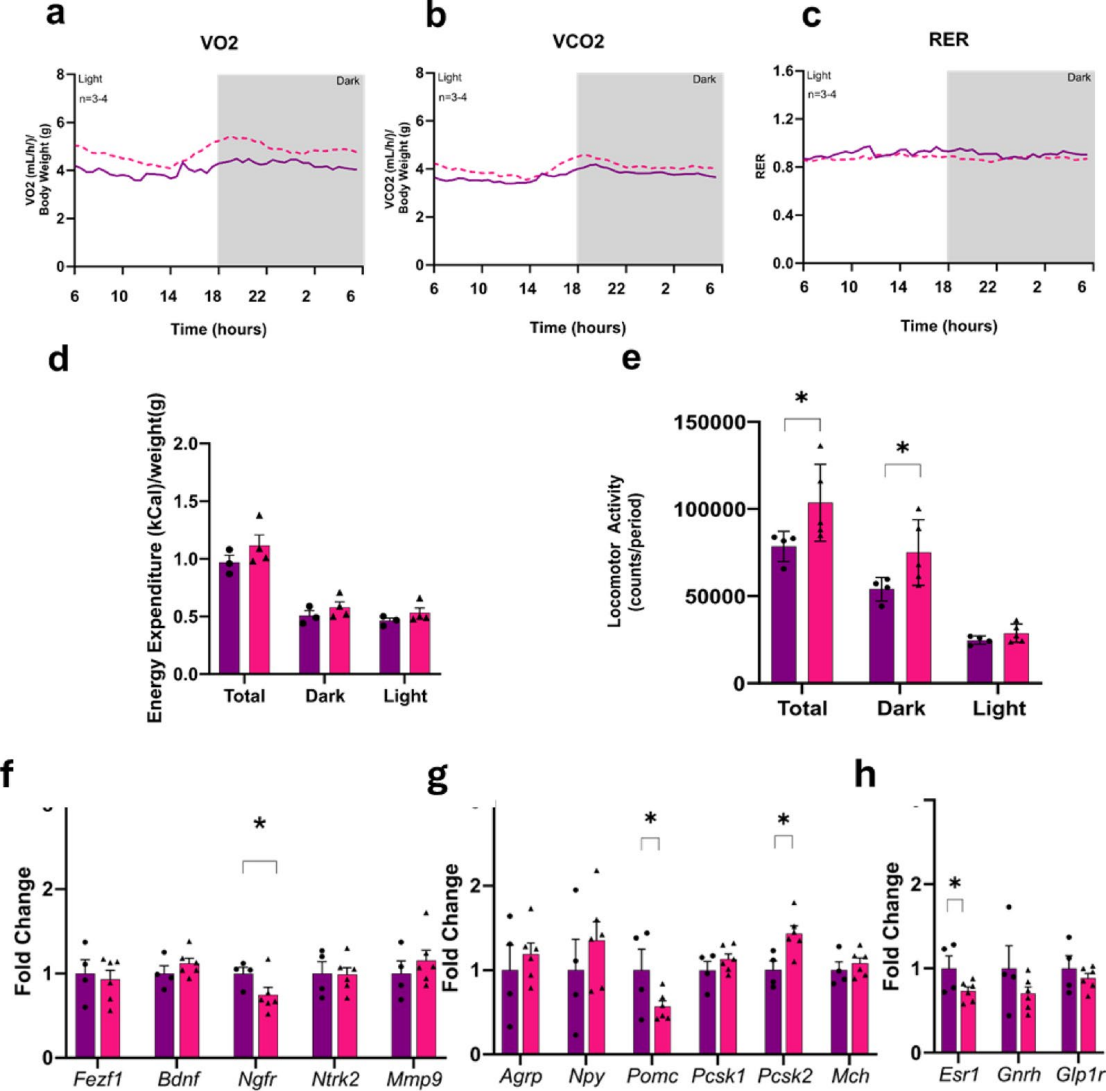
The outcomes of knocking BDNF out of Fezf1 neurons on energy expenditure and gene expression in female mice fed on a high-fat diet. All experiments were performed with Fezf1-cre/EGFP-L10a∆Bdnf (Fezf1-Bdnf knockout) female mice. Mice were placed in a respirometry chamber and parameters were evaluated over a 24-h period. Oxygen consumption (**a**), carbon dioxide production (**b**), respiratory exchange ratio (**c**), energy expenditure (**d**), and spontaneous locomotor activity (**e**). Transcript expression of hypothalamic genes involved in the Fezf1 and Bdnf systems (**f**). Transcript expression of hypothalamic genes involved in energy balance (**g**). Transcript expression of hypothalamic genes involved in reproduction and energy balance (**h**). Bdnf-flox (purple, Bdnfflox, control) and Fezf1-cre/EGFP-L10a∆Bdnf (pink, Fezf1-creDBdnf, Fezf1-Bdnf knockout). In all experiments, *n* = 4–6; *p < 0.05

*Energy expenditure in female Fezf1-Bdnf knockout mice fed on a HFD.* In female mice fed on a HFD, the knockout of BDNF in Fezf1 neurons was accompanied by increased oxygen consumption (Fig. 8a) and increased carbon dioxide production (Fig. 8b) with no difference in respiratory exchange ratio (Fig. 8c), resulting in a trend to increased energy expenditure (Fig. 8d). Mice under intervention presented increased spontaneous locomotor activity, particularly during the dark cycle (Fig. 8e).

*The impact of Fezf1-Bdnf knockout on hypothalamic gene expression.* As hypothalamic neurons are central players in the regulation of whole-body energy balance, we determined the transcript expression of hypothalamic genes involved in this function. In female mice fed on a HFD, the knockout of BDNF in Fezf1 neurons was accompanied by reductions in the hypothalamic expression of Ngfr (Fig. 8f), Pomc (Fig. 8g), and Esr1 (Fig. 8h); whereas the expression of Pcsk2 was increased (Fig. 8g). Several other hypothalamic genes involved in metabolic regulation were not affected by the intervention.

**Fig. 9:**
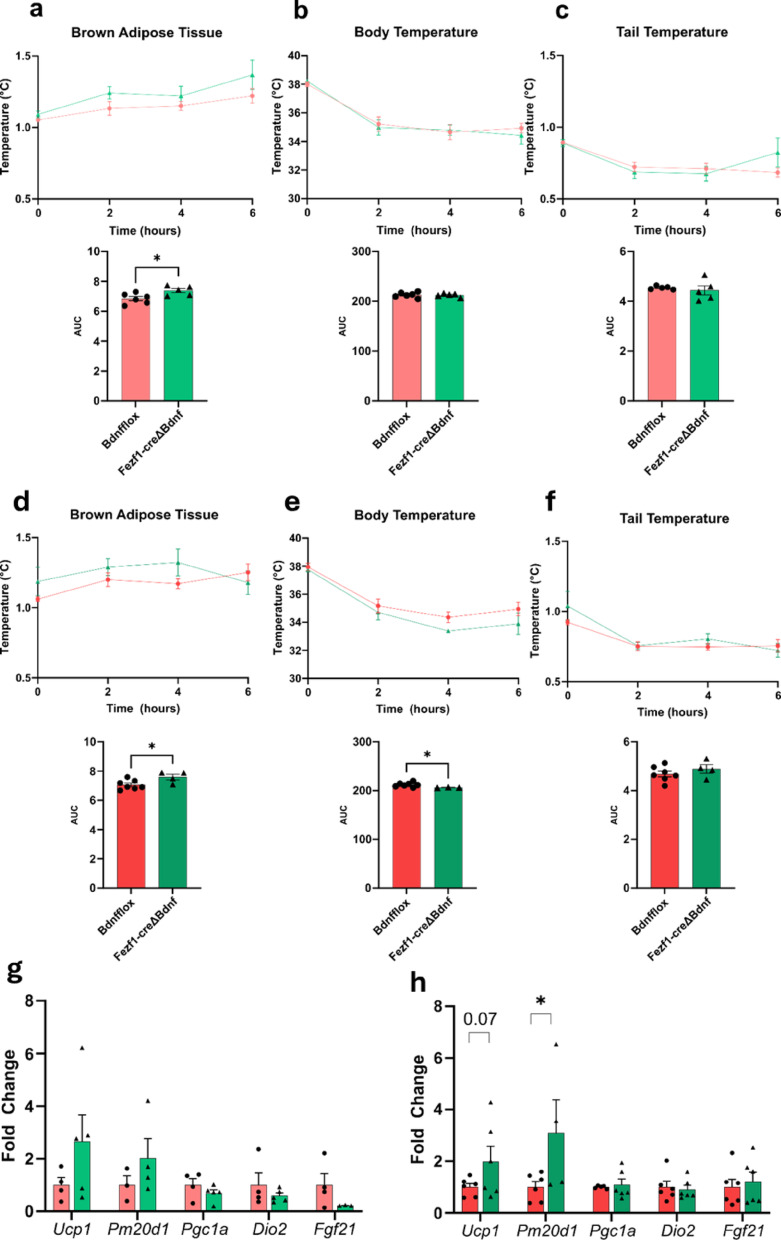
The impact of cold exposure in male mice submitted to the knockout of BDNF in Fexf1 neurons. All experiments were performed with Fezf1-cre/EGFP-L10a∆Bdnf (Fezf1-Bdnf knockout) male mice. Maximum interscapular (**a** and **d**), whole body (**b** and** e**), and tail (**c** and **f**) temperatures were determined in mice fed on chow (**a**-**c**) and fed on a high-fat diet (**d**-**f**); in each panel, the upper graph represents temperature over a period of six hours and the bottom panel represents the area under the temperature curve during the six hours recording. The transcript expressions of genes involved in thermoregulation were determined by quantitative real-time PCR in the brown adipose tissue of mice fed on chow (**g**) and a high-fat diet (**h**). In all experiments, *n* = 4–8; *p < 0.05

**Fig. 10:**
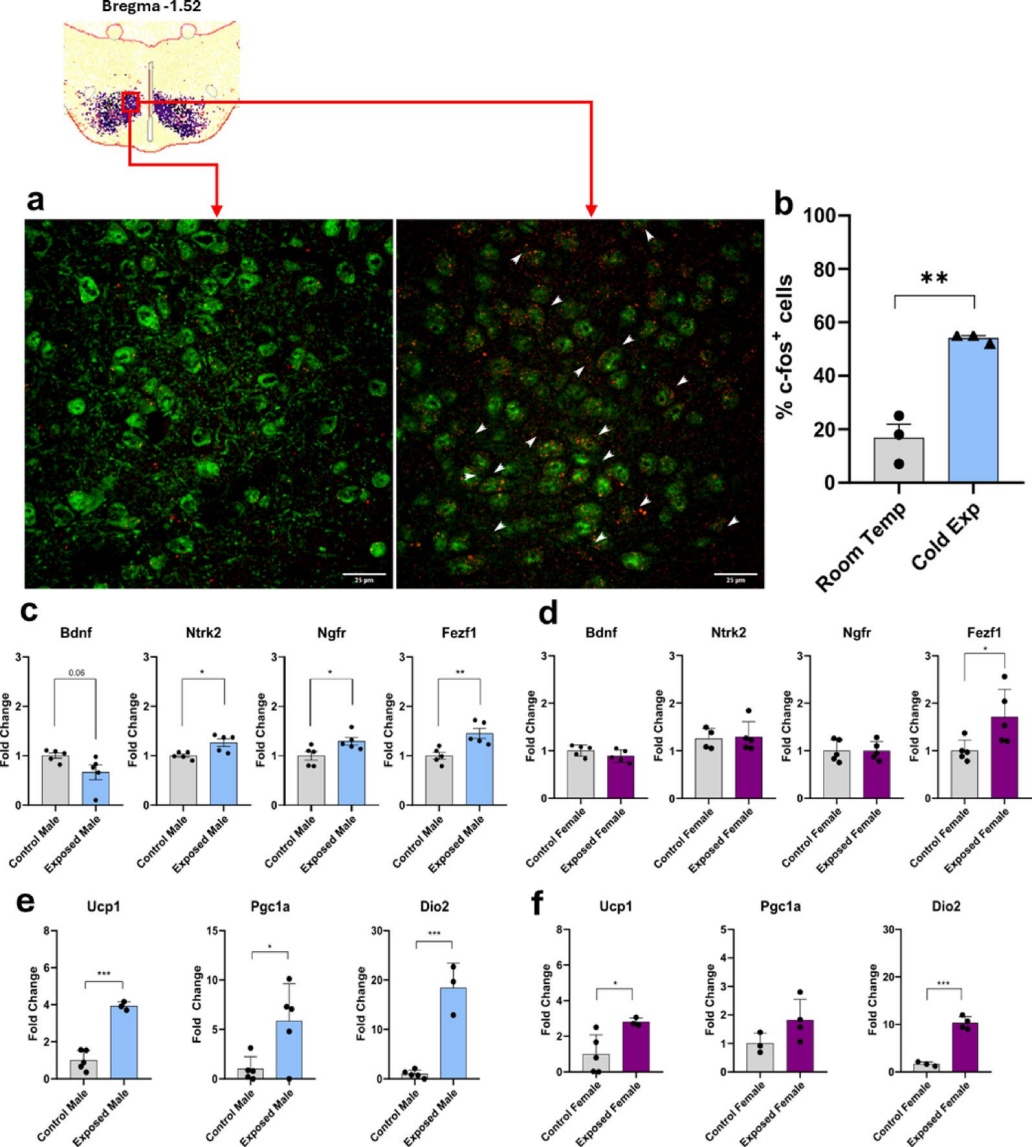
The impact of cold-exposure on c-fos expression in Fezf1 neurons. Fezf1 reporter mice were exposed to cold for 2-h, then the brains were harvested for determination of c-fos expression in the ventromedial hypothalamus at Bregma − 1.52 (**a**); the number of Fezf1 neurons expressing c-fos were counted (**b**). Hypothalamic expression of transcripts related to Fezf1 and Bdnf were determined by quantitative real-time PCR in males (**c**), and females (**d**). Brown adipose tissue expression of transcripts related to thermogenesis were determined by quantitative real-time PCR in males (**e**), and females (**f**). In all experiments, *n* = 3–5; *p < 0.05. In a, Fezf1, green; c-fos, red

*Increased cold-induced brown adipose tissue thermogenesis in male Fezf1-Bdnf knockout mice.* Cold exposure is a commonly used intervention that promotes fast metabolic adaptations in mammals. Here, we asked if the Fezf1-Bdnf knockout mice would respond differently than control to a short-term cold exposure intervention; for that, we measured body temperature variations and the BAT expression of genes involved in the regulation of thermogenesis. In female Fezf1-Bdnf knockout mice, an acute exposure to 4 °C promoted no changes in BAT mass and temperature, as well as no changes in body and tail temperature when fed either on chow (Suppl. Figure 14a-14c) or on a HFD (Suppl. Figure 15a-15c). Conversely, in males fed either on chow (Fig. 9a and c) or on a HFD (Fig. 9d and f), the knockout of Bdnf in Fezf1 neurons was accompanied by an increased BAT temperature, which was accompanied, in the mice fed on chow, by a trend to increase the expressions of Ucp1 and Pm20d1 (Fig. 9g), and in the mice fed on a HFD, by a trend to increase Ucp1 and a significant increase in Pm20d1 (Fig. 9h), nevertheless the BAT mass was not changed. Next, we asked if VMH neurons of Fezf1 mice are responsive to cold exposure. Thus, Fezf1 reporter mice were exposed to cold and then the brain was harvested for histological examination. As shown in Fig. 10a and b, the expression of c-fos was significantly increased in the Fezf1 neurons of mice exposed to cold. In male mice, this was accompanied by a reduction of Bdnf and an increase of Ntkr, Ngfr and Fezf1 (Fig. 10c); whereas in female mice, there were no changes in Bdnf, Ntkr and Ngfr, and an increase in Fezf1 (Fig. 10d). As a control for the effectiveness of the cold-exposure intervention, both male (Fig. 10e) and female (Fig. 10f) presented increased expressions of key thermogenic genes in the BAT.

## Discussion

This study has provided advance in the understanding of hypothalamic Fezf1 and BDNF biology by showing that: i, VMH Fezf1 neurons are an important source of BDNF in the hypothalamus; ii, Fezf1 neurons project to key regions involved in the regulation of metabolism and locomotor activity; iii, the knockdown of Bdnf specifically in Fezf1 neurons results in a sexual dimorphic phenotype characterized by the protection against diet-induced obesity in females and improved BAT thermogenesis in males. In addition, this study provides the first evidence that BDNF may have deleterious effects on metabolism when expressed in a particular cell subpopulation of the hypothalamus.

BDNF is widely expressed in the hypothalamus where it plays important roles in the regulation of neurogenesis, reproduction and metabolism [7, 9, 10, 12]. The VMH is the hypothalamic area with the greatest number of cells expressing BDNF, which are, at least in part, under the control of the melanocortinergic system [10]. Unfortunately, in most studies evaluating the roles of hypothalamic BDNF, the interventions aimed at modulating its actions lacked fine cellular specificity. Most studies in the field relied on injections of BDNF or non-cell-specific gene targeting. Thus, the injection of BDNF in the lateral ventricle had no impact on feeding and body mass of mice fed on chow, whereas in mice fed on a HFD it resulted in reduced caloric intake and reduced body mass gain [10]. The knockdown of Bdnf in SF1 neurons, which must be regarded as non-specific, since Sf1 is expressed in at least three distinct neuronal clusters [23], promoted hyperphagia and obesity [24]. The knockdown of Bdnf in neurons expressing Nkx2.1, which impacts a number of distinct neuronal subpopulations out of the VMH, resulted in a mild obese phenotype [24]. In addition, in studies using Bdnf-Cre mice, the inhibition of VMH Bdnf led to the identification of an ARC-VMH-perimesencephalic trigeminal area circuit that regulates caloric intake by gating motor sequences of feeding [25], and a leptin-dependent circuit that warrants proper sympathetic innervation of the adipose tissue [14]. Thus, taken together, the studies using non-cell-specific strategies to modulate the expression or action of hypothalamic Bdnf/BDNF suggests it exerts a metabolically positive action, reducing food intake, protecting against obesity and metabolic abnormalities, and increasing energy expenditure; however, it was previously unknown if this pattern is common for all cell-types expressing BDNF.

In the first part of this study, we re-analyzed data from a single-nucleus transcriptomics [16] with the objective of providing a fine mapping of the neuronal identities that express BDNF in the hypothalamus. Fezf1 neurons emerged as the second most important source of VMH BDNF, and, since very little is known about the roles of Fezf1 neurons in adult life, we decided to intervene in this subpopulation.

In addition to Fezf1, Bdnf transcripts were also present in Dlk1, Lepr, Foxp2, Esr1 and Nfib neurons of the VMH. Mutations in the Dlk1 gene result in precocious puberty and obesity [26]; however, it is currently unknown if this phenotype is due to abnormalities in the functions of VMH DLK1 neurons, and if BDNF is somehow involved in the pathophysiology of the diseases. Of course, considering that VMH neurons expressing Dlk1 were those co-expressing Bdnf in the greatest amount, studies should focus on this particular subset of neurons to further explore the role of Bdnf in the hypothalamus. Lepr is widely expressed in the VMH, and single-nucleus transcriptomics have identified at least nine subtypes of Lepr-expressing neurons in this region [16]. The injection of leptin in the VMH promotes an increase in the expression of BDNF; however, it is unknown what are the functions of BDNF expressed by these particular subsets of neurons. There is no data linking hypothalamic BDNF with Foxp2, Esr1 or Nfib. Thus, our data expands the understanding about the subpopulations of cells expressing Bdnf and provides a wide range of opportunities for exploring the actions of BDNF expressed in distinct subsets of VMH neurons.

As very little was known regarding the distribution and projections of Fefzf1 neurons, we employed three distinct strategies to provide advance in this field. First, we used the Allen Brain Atlas to determine the whole brain distribution of the Fezf1 neurons. We showed that, in the adult mouse, Fezf1 neurons are virtually restricted to the VMH and striatum. This implies that our strategy of knocking down Bdnf specifically in Fezf1 neurons, affected mostly the VMH population, which is much greater than the striatum, but it does not rule out that, at least some of the phenotype resulting from the intervention, could be due to reduction of Bdnf in the striatum population. Next, we employed a Fezf1 reporter mouse, which confirmed the predominant presence of Fezf1 neurons in the VMH and the co-expression with BDNF. We also showed that VMH Fezf1 neurons express SF1, an important marker of the VMH neurons [27], which is involved in the expression of gonadotrophic factors [28], and may explain the sexual dimorphic phenotypes of our model [29]. Another relevant finding is that Fezf1 neurons express the receptor for GLP-1. The studies exploring the mechanisms behind body mass reduction obtained using GLP-1 receptor agonists identified hypothalamic sites of action for this class of drugs, and our results may expand the knowledge about the central actions of liraglutide, semaglutide and the new single-, dual-, and triple-agonists entering the market for treating patients with obesity [30–32]. As a third approach to evaluate the anatomical particularities of the VMH Fezf1 neurons, we used a viral delivered tracer that evaluated the projections originating from the VMH Fezf1 neurons; we showed that, within the hypothalamus there are projections to the DMH and ZI, whereas out of the hypothalamus there are projections to the periaqueductal gray matter, amygdala, and hippocampal regions CA1 and CA3. The DMH is involved in the regulation of systemic metabolic function and acts as an interface between metabolism and circadian behavior [33]. Thus, at least in part, these projections may be involved in the metabolic phenotype presented by our model. The ZI is involved in motricity and locomotor activity [34], and in our model, female mice fed on a HFD presented increased locomotor activity, which can be regarded as one of the mechanisms behind the protection against diet-induced obesity. The connections to the periaqueductal gray matter may be involved in the output of autonomic signals that have been previously described in studies evaluating VMH BDNF [14, 35]. To our knowledge, no prior studies have explored the connectivity between BDNF-expressing neurons of the VMH and the amygdala and hippocampus.

Next, we obtained Fezf1-specific knockout of Bdnf by crossing Fezf1-cre (B6.Cg-Fezf1tm1.1(cre/folA)Hze/J) with Bdnf-lox (B6J.129S4-Bdnftm3Jae/RujfJ) mice. The conditional deletion of BDNF was induced in the sixth week of life, therefore avoiding potential developmental problems that could emerge from a constitutive knockout. In both, female and male mice fed on chow, the intervention resulted in no change in the metabolic phenotype, indicating that Fezf1-Bdnf has no important role in the regulation of systemic metabolism in young adult mice fed on an equilibrated diet. However, when mice were fed on a HFD, metabolic phenotypes emerged in both female and male; nevertheless, despite the fact that in either sex the metabolic outcomes were dysfunctional, they differed in nature. In females there was a protection against diet-induced obesity, due to reduced caloric intake and increased locomotor activity, whereas in males there was an increase in BAT thermogenic response to cold exposure.

In the females, the phenotype included improved glucose tolerance and increased insulin sensitivity. The VMH has been long known as an important site of regulation of systemic glucose levels. Early studies that evaluated rodents submitted to lesions of the VMH, reported the development of glucose intolerance and insulin resistance [36, 37]. These findings were confirmed in several studies that specifically targeted distinct types of VMH neurons and components of the signaling systems that regulate their activity [38–41]. Thus, our data provides advance in the field by showing that, at least in part, BDNF expressed specifically in Fezf1 neurons plays an important role in the regulation of glucose metabolism.

The increased locomotor activity was yet another important component of the phenotype presented by the females. In prior studies that performed VMH lesions, there were reductions in fasting-induced locomotor activity [42, 43], whereas in approaches targeting the SF1 neurons of the VMH, authors reported an integration of metabolic and locomotor signals, which was interpreted as a key component of the feeding-behavioral VMH centered network [44]. Moreover, in a study targeting the BDNF receptor TrkB, there was a phenotype of reduced locomotor activity that predominated in females [45]. Our data adds important information, confirming the role of VMH neurons on the control of metabolic-associated locomotor activity and showing that Fezf1-BDNF provides a sex-dependent regulation of this function.

In males, the phenotype was related to cold induced activation of BAT thermogenesis. Interestingly, in early studies, which were mostly performed in male rodents, the stimulation of the VMH resulted in increased BAT thermogenesis [46, 47]. However, later on, when females were analyzed, a sexual dimorphism related to VMH control of BAT activity was reported [48]. In our study, the exposure of wildtype mice to cold promoted a rapid increase in the expression of the early-responsive gene, c-fos, in the Fezf1 neurons, and this was accompanied by increased expression of the BDNF receptors, Trkb and Ngfr, in males, only. In male Fezf1-Bdnf knockdown mice, the increased cold-induced BAT thermogenesis was accompanied by increased Ucp1 and Pm20d1. UCP1 provides the most important and most widely studied mechanism of BAT thermogenesis [49]. However, studies have identified three other, UCP1-independent, mechanisms behind the induction of thermogenesis in the BAT: i, calcium influx [50]; ii, creatine cycle [51]; and, iii, PM20D1 and N-acyl amino acids [52]. PM20D1 is secreted by brown adipocytes catalyzing the synthesis of N-acyl amino acids from unsaturated fatty acids and amino acids. Once produced, the N-acyl amino acids enter the brown adipocytes, directly binding to mitochondria, and promoting UCP1-independent uncoupling and the release of heat [52]. Thus, our data suggests that, in males, Fezf1-Bdnf can control BAT activity by UCP1-dependent, and at least one UCP1-independent mechanisms.

It is important to mention that prior studies have evaluated the impact of sex on the expression of either Fezf1 or Bdnf in the hypothalamus. The expression pattern of Fezf1 does not differ between males and females, and the estrous cycle does not affect its expression. In contrast, there are differences in Bdnf expression between males and females, with estrogen increasing its hypothalamic expression [53–56]. However, all those studies evaluated the genes, transcripts, or proteins separately. Here, we provide the first combined evaluation of Fezf1 and Bdnf.

An unexpected finding of our study was the fact that knocking out Bdnf from VMH-Fezf1 neurons resulted in positive metabolic outcomes. Most studies published to date show that interventions aimed at modulating hypothalamic Bdnf/BDNF resulted in improved metabolic phenotype when BDNF was increased, whereas the opposite phenotype occurred when BDNF was reduced [13, 15, 57]. However, there are some studies showing that under certain conditions, BDNF may have detrimental effects. That is the case for a nucleus of the solitary tract-VMH pathway that controls sympathetic outflow to regulate the blood pressure, in which, increased VMH BDNF results in increased blood pressure [58]. In addition, VMH BDNF was negatively involved in stress-induced hypertension [59]. The role of BDNF on ageing, is also controversial as a number of studies have shown that increased BDNF may be found in Alzheimer’s Disease and conditions leading to accelerated biological ageing [60, 61]. Thus, our findings reveal a specific condition where reduction of Bdnf may have beneficial implications in metabolism.

In conclusion, our study provides advance in understanding how the VMH is involved in the regulation of whole-body metabolism. For the first time, it was shown that Fezf1 neurons play an important role in adult-life metabolism in a sexual dimorphic way. In addition, we showed that Bdnf may have unexpected detrimental effects on metabolism when expressed in a particular VMH neuron subpopulation.

## Supplementary Information

Supplementary Material 1

## Data Availability

Data will be available upon request directed to the corresponding author, LAV (* [*lavellos@unicamp.br*](mailto: lavellos@unicamp.br) *).
