# Supplementary material for: Metabolic sexual dimorphism in hypothalamic Fezf1 neuron-specific BDNF knockout
Source: Biol Sex Differ. 2025 Nov 11;16:95. doi: 10.1186/s13293-025-00770-z (PMC12606896; doi:10.1186/s13293-025-00770-z)

Supplementary data

Metabolic sexual dimorphism in hypothalamic Fezf1 neuron-specific BDNF knockout

Dayana Cabral-da-Silva^1^, Ariane M. Zanesco^1^, Fernando Valdivieso-Rivera^1^, Ana L. Gallo-Ferraz^1^, Marcela R. Simões^1^, Bruna Bombassaro^1^, Carlos H. Sponton^1,2^, Licio A. Velloso^1,3,*^

**Supplementary Figure 1. Determination of the co-expression of Fezf1 versus Sf1 or Glipr1.** In the hypothalamus, 63% of Fezf1 positive cells co-express Sf1 (a), whereas 86% co-express Glipr1, gene coding for the GLP1 receptor (b). Measurements were performed using the public hypothalamic single-cell transcriptomic dataset from the Chan Zuckenberg Initiative CellxGene plataform (https://cellxgene.cziscience.com).

**
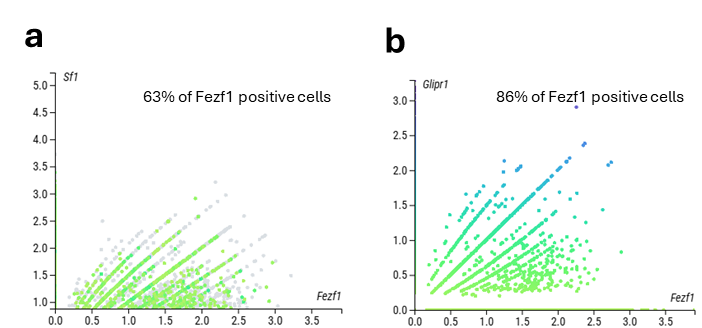
**

**Supplementary Figure 2. The outcomes of knocking BDNF out of Fezf1 neurons on body mass and food intake in male mice fed on chow.** Weekly determination of body weight (a), area under the curve (AUC) of body weight gain (b), and cumulative food intake over a period of nine weeks (c). Bdnf-flox (red, Bdnfflox, control) and Fezf1-cre/EGFP-L10a∆Bdnf (green, Fezf1-creΔBdnf, Fezf1-Bdnf knockout). In all experiments, n=5-7.


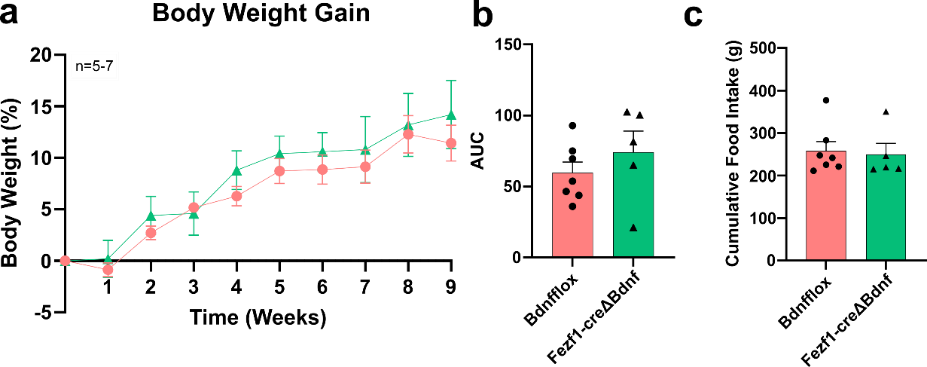


**Supplementary Figure 3. The outcomes of knocking BDNF out of Fezf1 neurons on glucose tolerance in male mice fed on chow.** Blood levels of glucose in fasting mice (a); graphic representation of blood glucose levels during a glucose-tolerance test (B); area under the curve (AUC) obtained from the blood glucose level variations during the glucose tolerance test (c). Bdnf-flox (red, Bdnfflox, control) and Fezf1-cre/EGFP-L10a∆Bdnf (green, Fezf1-creΔBdnf, Fezf1-Bdnf knockout). In all experiments, n=5.


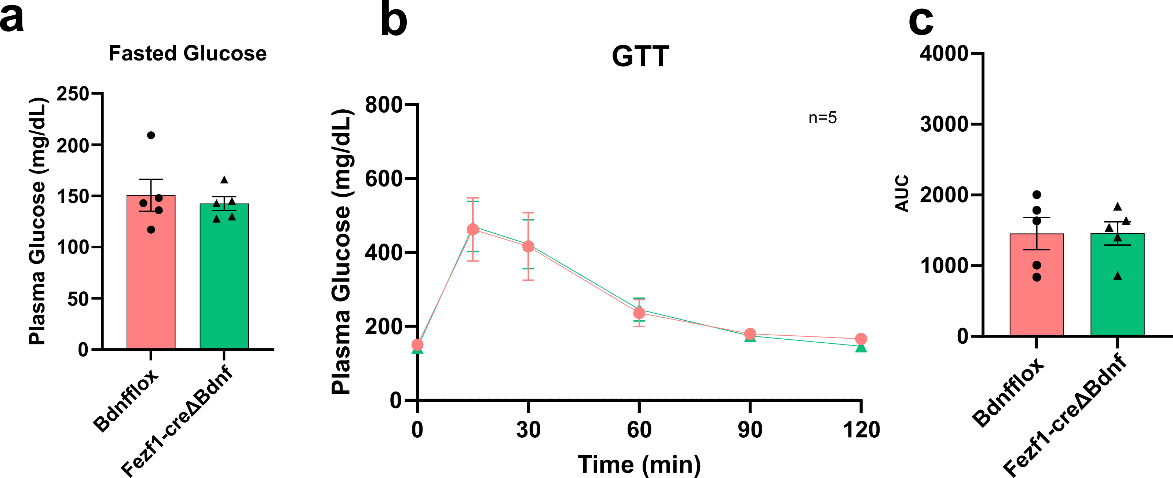


**Supplementary Figure 4. The outcomes of knocking BDNF out of Fezf1 neurons on insulin sensitivity in male mice fed on chow.** Blood levels of glucose in fasting mice (a); graphic representation of blood glucose levels during an insulin tolerance test (B); graphic representation of the constant of the blood glucose disappearance rate (kITT) obtained from the blood glucose level variations during the insulin tolerance test (c). Bdnf-flox (red, Bdnfflox, control) and Fezf1-cre/EGFP-L10a∆Bdnf (green, Fezf1-creΔBdnf, Fezf1-Bdnf knockout). In all experiments, n=5-6.


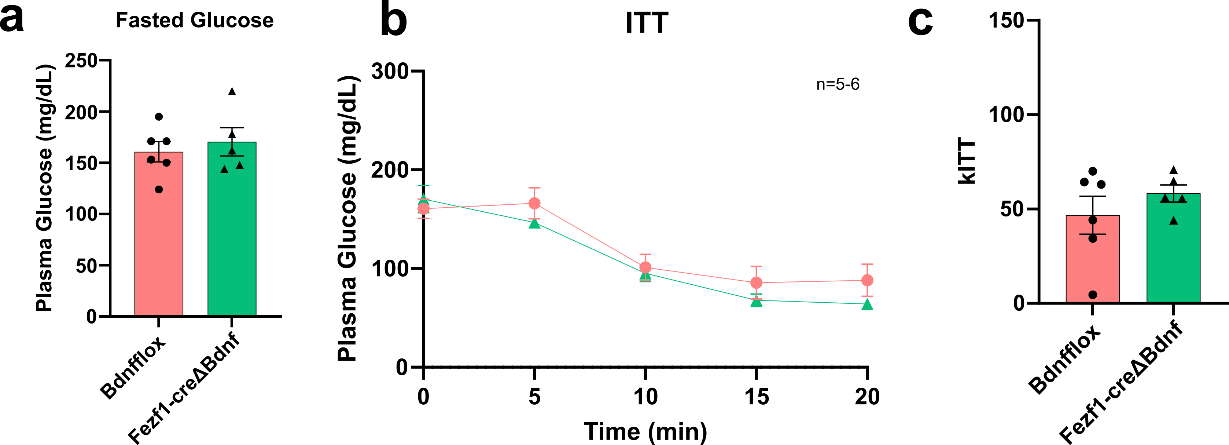


**Supplementary Figure 5. The outcomes of knocking BDNF out of Fezf1 neurons on energy expenditure in male mice fed on chow.** Mice were placed in a respirometry chamber and parameters were evaluated over a 24-h period. Oxygen consumption (a), carbon dioxide production (b), respiratory exchange ratio (c), energy expenditure (d), and spontaneous locomotor activity (e). Bdnf-flox (red, Bdnfflox, control) and Fezf1-cre/EGFP-L10a∆Bdnf (green, Fezf1-creΔBdnf, Fezf1-Bdnf knockout). In all experiments, n=4-5.


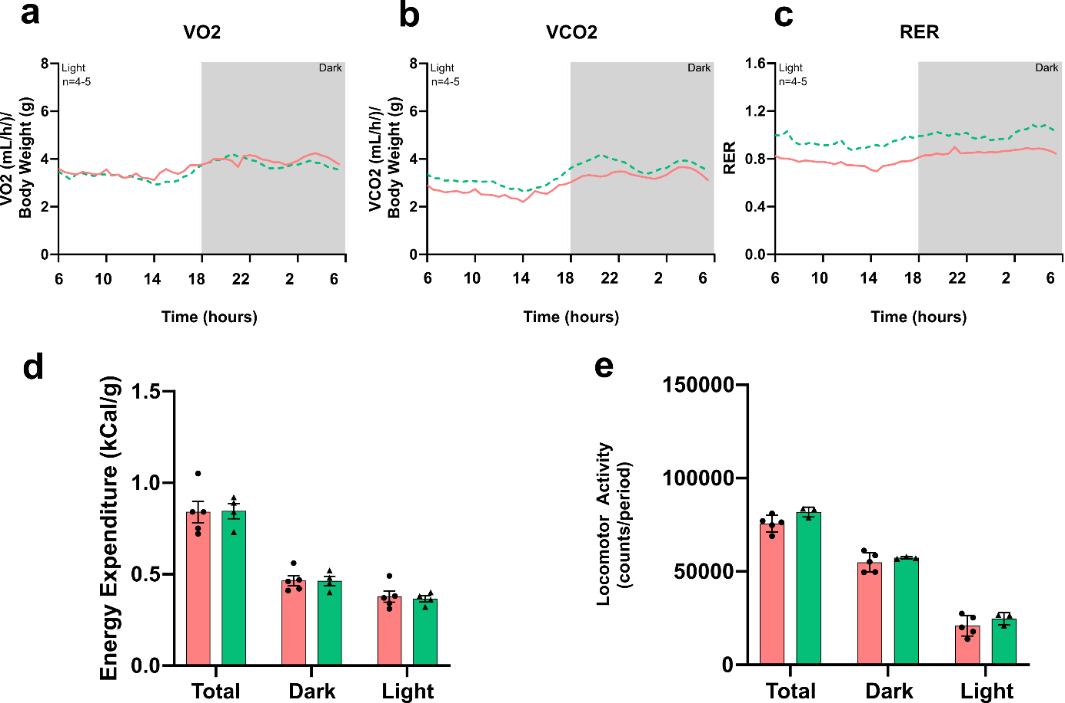


**Supplementary Figure 6. The outcomes of knocking BDNF out of Fezf1 neurons on body mass and food intake in male mice fed on a high-fat diet.** Weekly determination of body weight (a), area under the curve (AUC) of body weight gain (b), and cumulative food intake over a period of nine weeks (c). Bdnf-flox (red, Bdnfflox, control) and Fezf1-cre/EGFP-L10a∆Bdnf (green, Fezf1-creΔBdnf, Fezf1-Bdnf knockout). In a, the introduction of a high-fat diet (HFD) is indicated with an arrow. In all experiments, n=8.


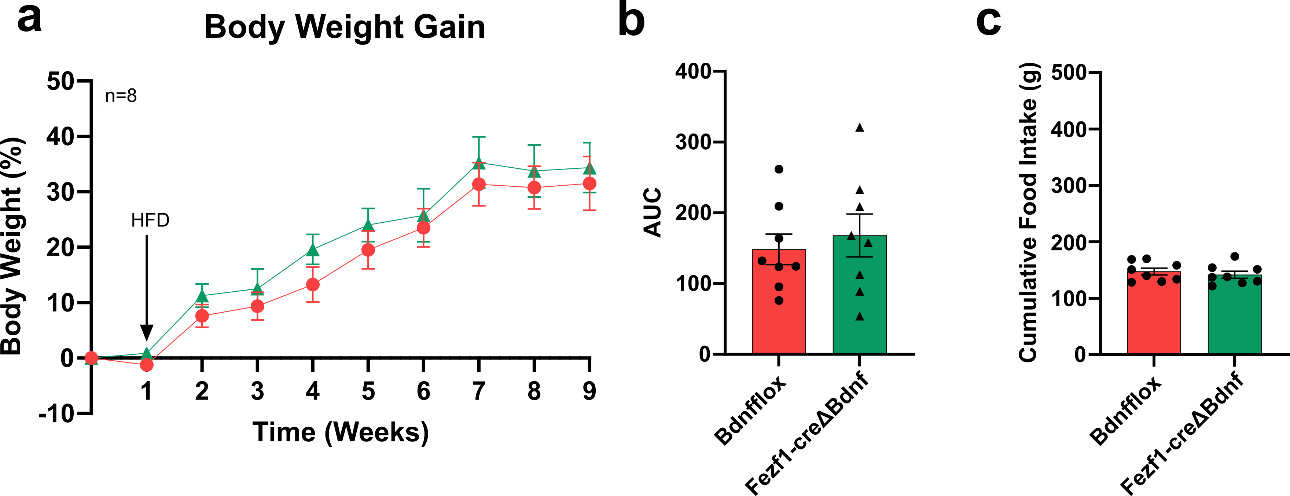


**Supplementary Figure 7. The outcomes of knocking BDNF out of Fezf1 neurons on glucose tolerance in male mice fed on a high-fat diet.** Blood levels of glucose in fasting mice (a); graphic representation of blood glucose levels during a glucose-tolerance test (B); area under the curve (AUC) obtained from the blood glucose level variations during the glucose tolerance test (c). Bdnf-flox (red, Bdnfflox, control) and Fezf1-cre/EGFP-L10a∆Bdnf (green, Fezf1-creΔBdnf, Fezf1-Bdnf knockout). In all experiments, n=6.


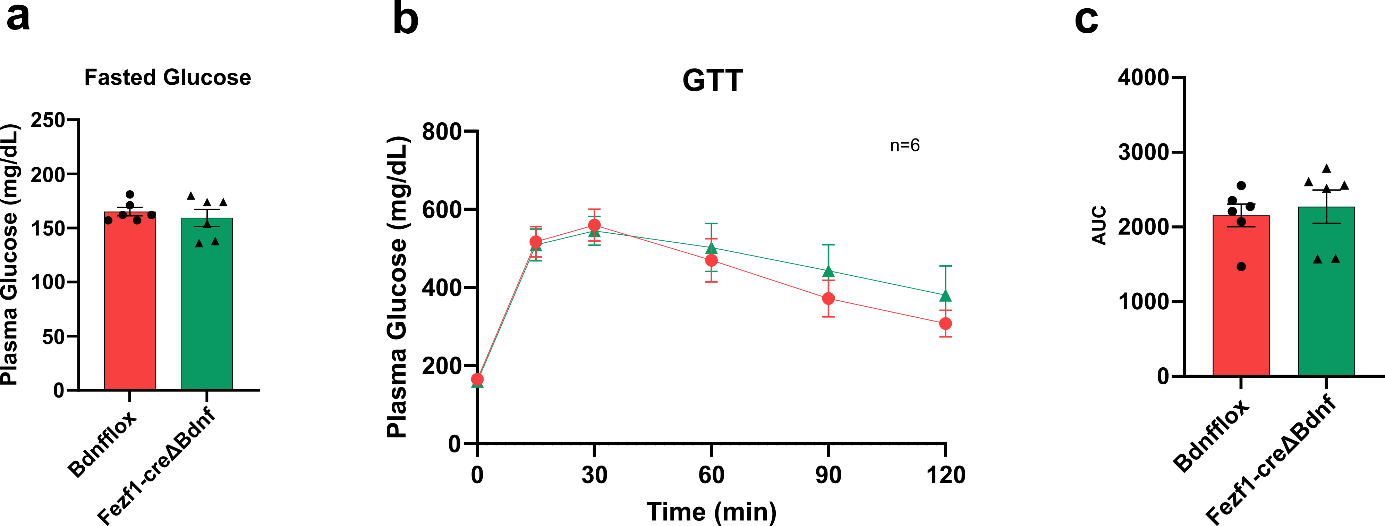


**Supplementary Figure 8. The outcomes of knocking BDNF out of Fezf1 neurons on insulin sensitivity in male mice fed on a high-fat diet.** Blood levels of glucose in fasting mice (a); graphic representation of blood glucose levels during an insulin tolerance test (B); graphic representation of the constant of the blood glucose disappearance rate (kITT) obtained from the blood glucose level variations during the insulin tolerance test (c). Bdnf-flox (red, Bdnfflox, control) and Fezf1-cre/EGFP-L10a∆Bdnf (green, Fezf1-creΔBdnf, Fezf1-Bdnf knockout). In all experiments, n=6-7.


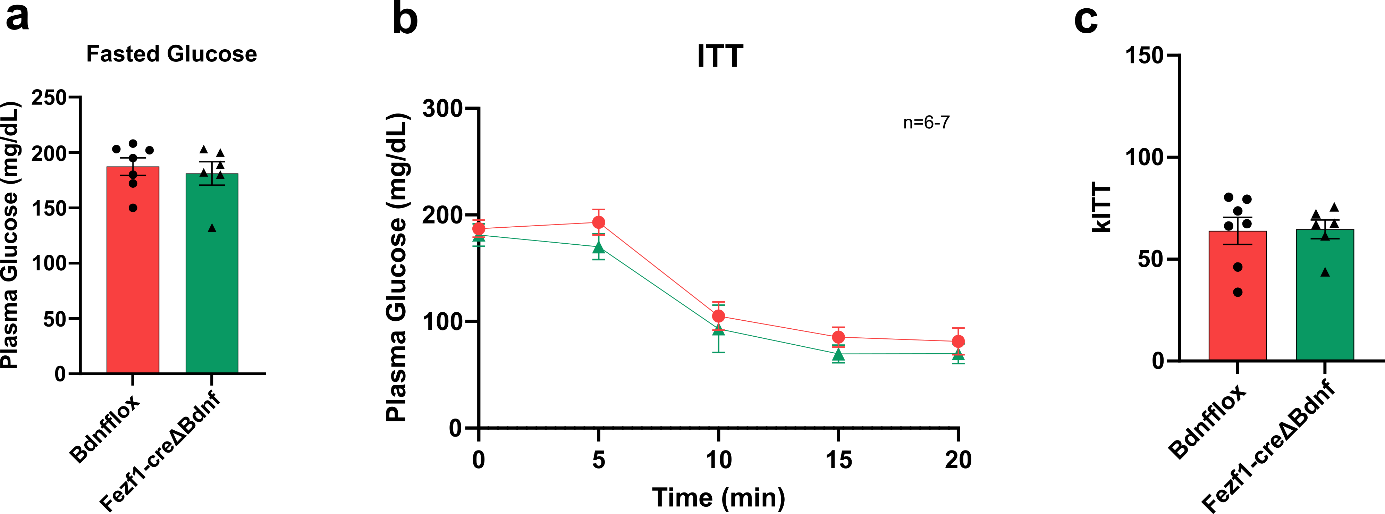


**Supplementary Figure 9. The outcomes of knocking BDNF out of Fezf1 neurons on energy expenditure in male mice fed on a high-fat diet.** Mice were placed in a respirometry chamber and parameters were evaluated over a 24-h period. Oxygen consumption (a), carbon dioxide production (b), respiratory exchange ratio (c), energy expenditure (d), and spontaneous locomotor activity (e). Bdnf-flox (red, Bdnfflox, control) and Fezf1-cre/EGFP-L10a∆Bdnf (green, Fezf1-creΔBdnf, Fezf1-Bdnf knockout). In all experiments, n=5.


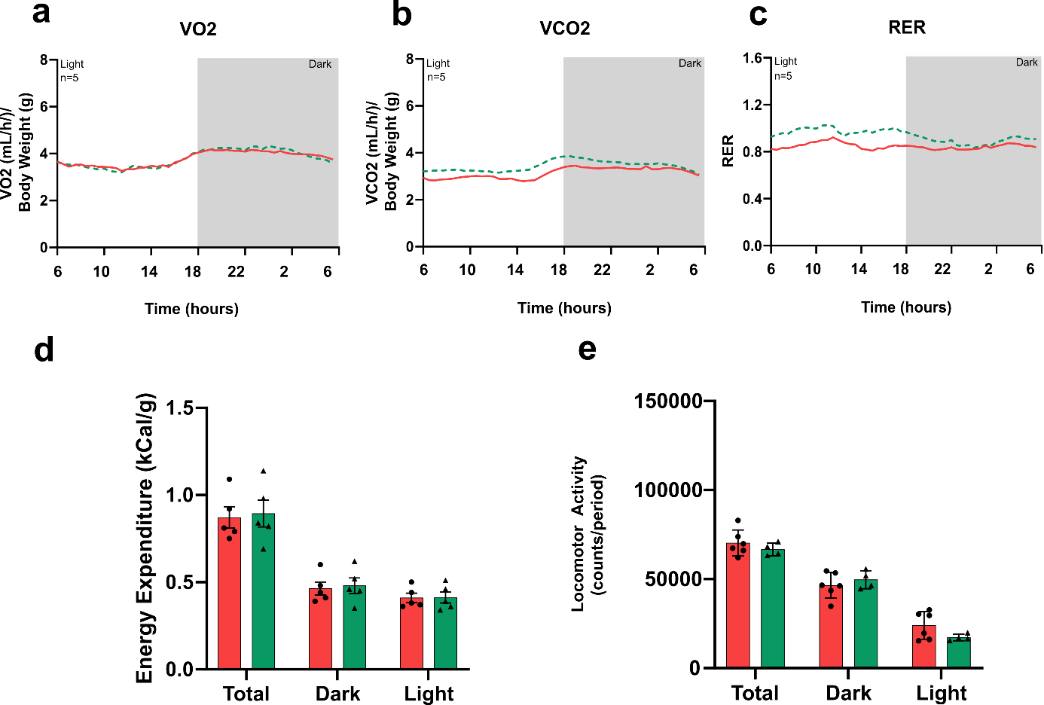


**Supplementary Figure 10. The outcomes of knocking BDNF out of Fezf1 neurons on body mass and food intake in female mice fed on chow.** Weekly determination of body weight (a), area under the curve (AUC) of body weight gain (b), and cumulative food intake over a period of nine weeks (c). Bdnf-flox (violet, Bdnfflox, control) and Fezf1-cre/EGFP-L10a∆Bdnf (pink, Fezf1-creΔBdnf, Fezf1-Bdnf knockout). In all experiments, n=6.


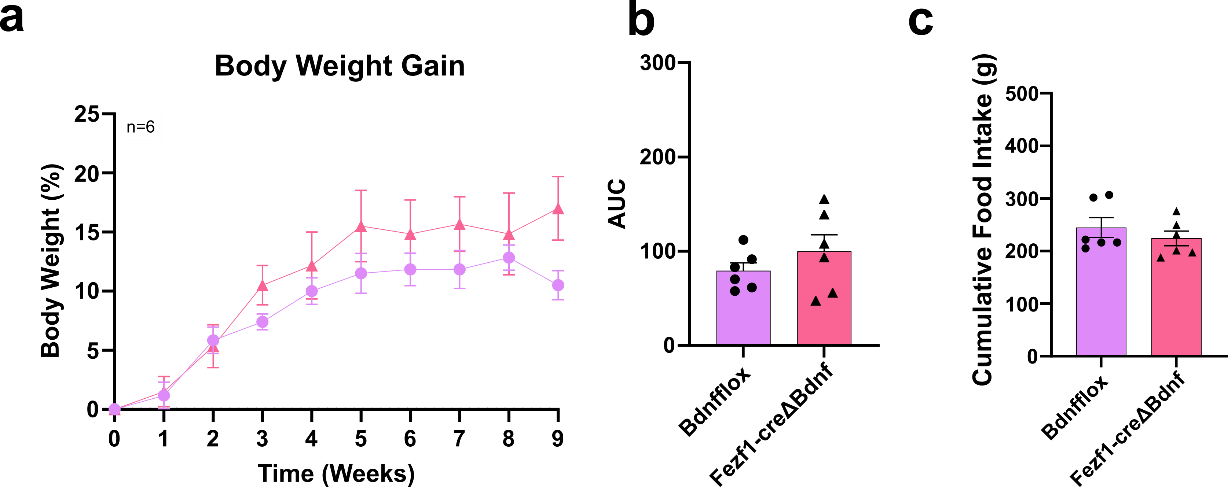


**Supplementary Figure 11. The outcomes of knocking BDNF out of Fezf1 neurons on glucose tolerance in female mice fed on chow.** Blood levels of glucose in fasting mice (a); graphic representation of blood glucose levels during a glucose-tolerance test (B); area under the curve (AUC) obtained from the blood glucose level variations during the glucose tolerance test (c). Bdnf-flox (violet, Bdnfflox, control) and Fezf1-cre/EGFP-L10a∆Bdnf (pink, Fezf1-creΔBdnf, Fezf1-Bdnf knockout). In all experiments, n=6.


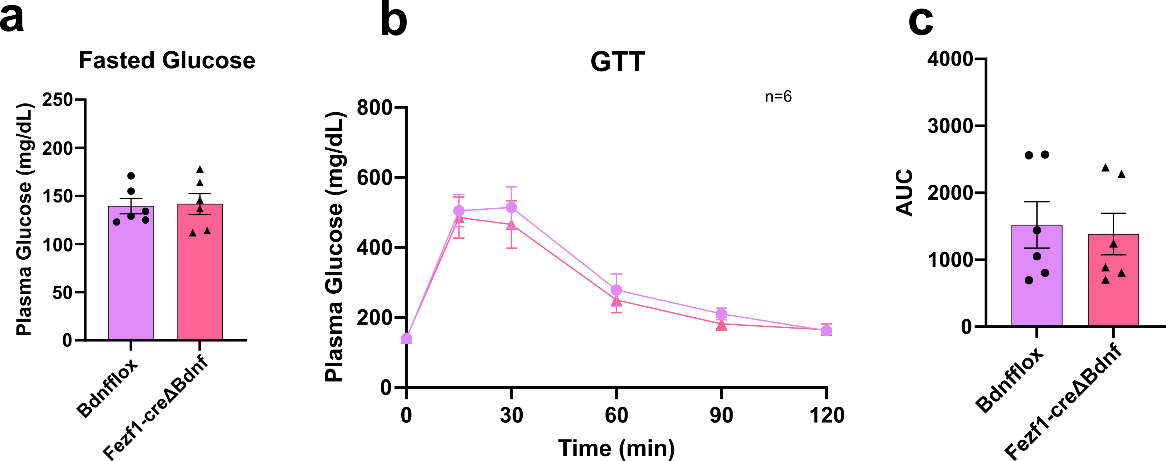


**Supplementary Figure 12. The outcomes of knocking BDNF out of Fezf1 neurons on insulin sensitivity in female mice fed on chow.** Blood levels of glucose in fasting mice (a); graphic representation of blood glucose levels during an insulin tolerance test (B); graphic representation of the constant of the blood glucose disappearance rate (kITT) obtained from the blood glucose level variations during the insulin tolerance test (c). Bdnf-flox (violet, Bdnfflox, control) and Fezf1-cre/EGFP-L10a∆Bdnf (pink, Fezf1-creΔBdnf, Fezf1-Bdnf knockout). In all experiments, n=4.


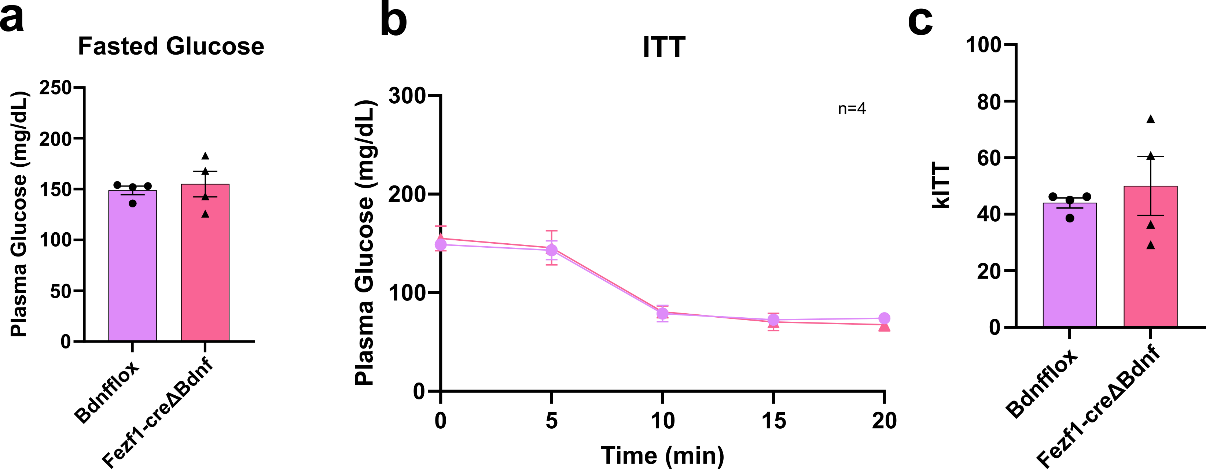


**Supplementary Figure 13. The outcomes of knocking BDNF out of Fezf1 neurons on energy expenditure in female mice fed on chow.** Mice were placed in a respirometry chamber and parameters were evaluated over a 24-h period. Oxygen consumption (a), carbon dioxide production (b), respiratory exchange ratio (c), energy expenditure (d), and spontaneous locomotor activity (e). Bdnf-flox (violet, Bdnfflox, control) and Fezf1-cre/EGFP-L10a∆Bdnf (pink, Fezf1-creΔBdnf, Fezf1-Bdnf knockout). In all experiments, n=5.


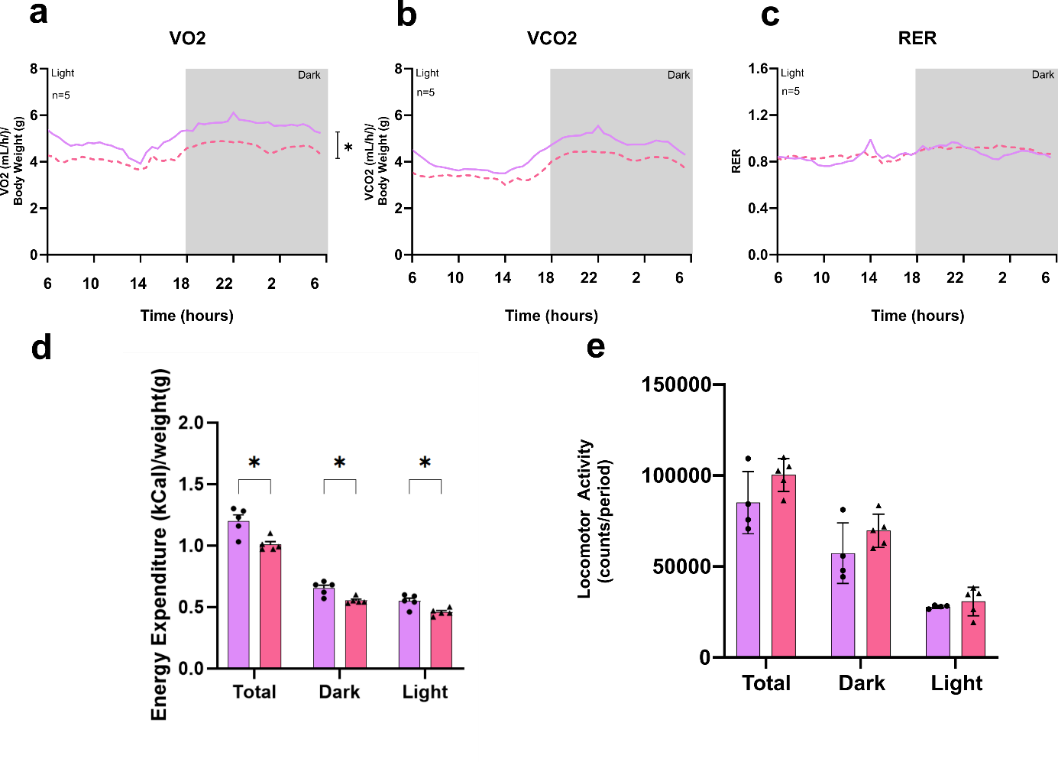


**Supplementary Figure 14. The outcomes of knocking BDNF out of Fezf1 neurons on the thermogenic response in female mice fed on chow and exposed to cold.** Curves and area under the curves for interscapular (a), body (b) and tail (c) temperatures of mice exposed to +4^o^C for 6 h. Bdnf-flox (violet, Bdnfflox, control) and Fezf1-cre/EGFP-L10a∆Bdnf (pink, Fezf1-creΔBdnf, Fezf1-Bdnf knockout). In all experiments, n=5.


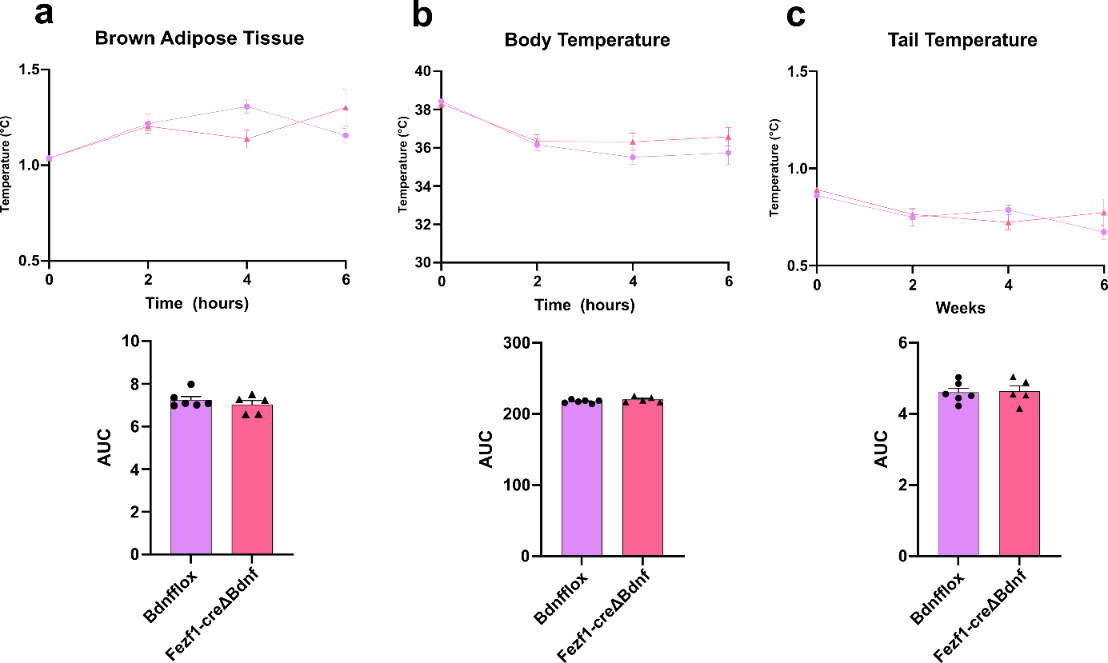


**Supplementary Figure 15. The outcomes of knocking BDNF out of Fezf1 neurons on the thermogenic response in female mice fed on a high-fat diet and exposed to cold.** Curves and area under the curves for interscapular (a), body (b) and tail (c) temperatures of mice exposed to +4^o^C for 6 h. Bdnf-flox (purple, Bdnfflox, control) and Fezf1-cre/EGFP-L10a∆Bdnf (pink, Fezf1-creΔBdnf, Fezf1-Bdnf knockout). In all experiments, n=5.


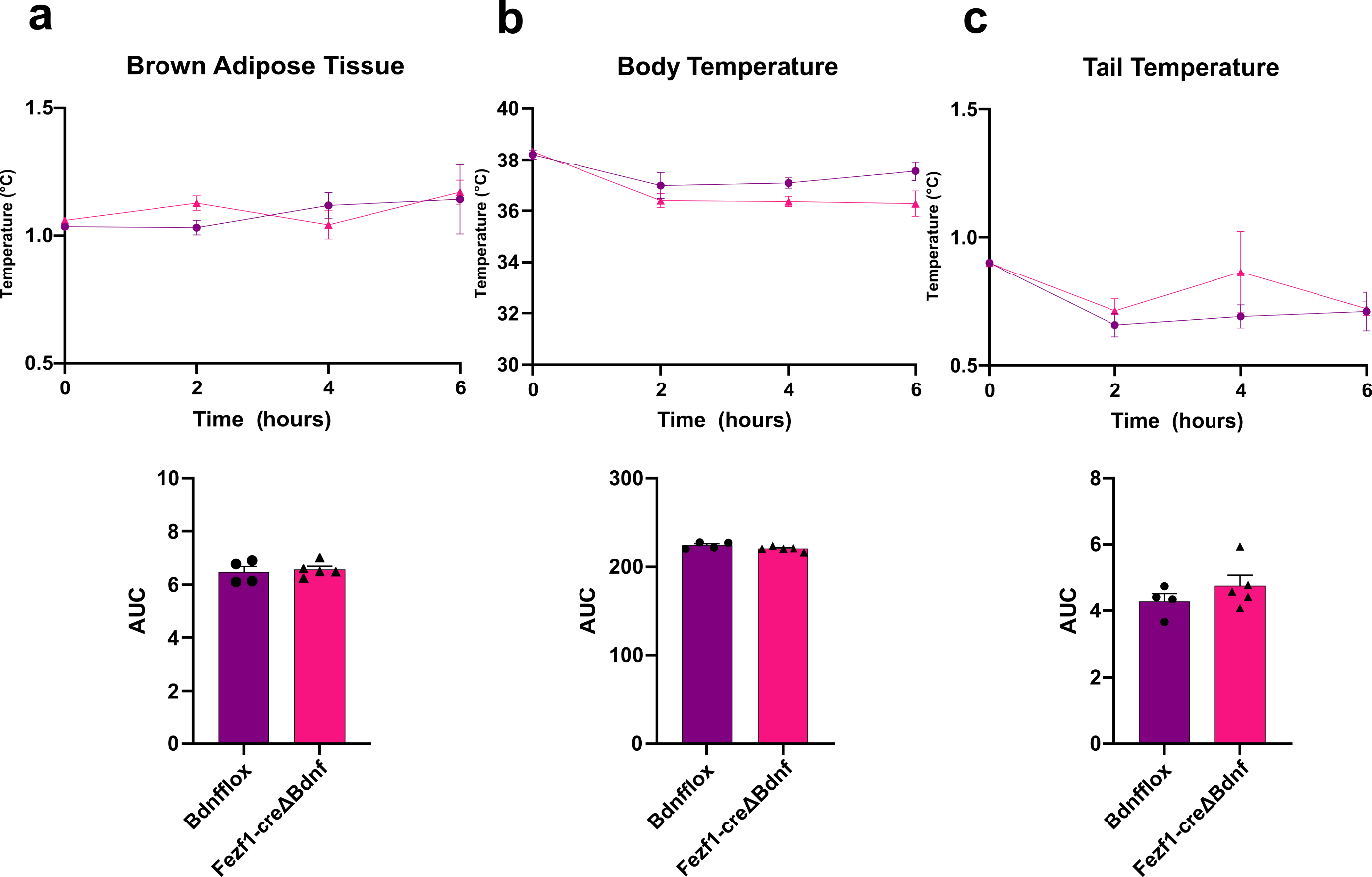

Supplement: Supplementary file 1 — Supplementary Material 1 [file 13293_2025_770_MOESM1_ESM.docx]
